# The impact of schizophrenia genetic load and heavy cannabis use on the risk of psychotic disorder in the EU-GEI case-control and UK Biobank studies

**DOI:** 10.1017/S0033291724002058

**Published:** 2024-11

**Authors:** Isabelle Austin-Zimmerman, Edoardo Spinazzola, Diego Quattrone, Beatrice Wu-Choi, Giulia Trotta, Zhikun Li, Emma Johnson, Alexander L. Richards, Tom P. Freeman, Giada Tripoli, Charlotte Gayer-Anderson, Victoria Rodriguez, Hannah E. Jongsma, Laura Ferraro, Caterina La Cascia, Sarah Tosato, Ilaria Tarricone, Domenico Berardi, Elena Bonora, Marco Seri, Giuseppe D'Andrea, Andrei Szöke, Celso Arango, Julio Bobes, Julio Sanjuán, Jose Luis Santos, Manuel Arrojo, Eva Velthorst, Miguel Bernardo, Cristina Marta Del-Ben, Paulo Rossi Menezes, Jean-Paul Selten, Peter B. Jones, James B. Kirkbride, Bart P. F. Rutten, Andrea Tortelli, Pierre-Michel Llorca, Lieuwe de Haan, Simona Stilo, Daniele La Barbera, Antonio Lasalvia, Franck Schurnhoff, Baptiste Pignon, Jim van Os, Michael Lynskey, Craig Morgan, EU-GEI WP2 Group, Michael O’ Donovan, Cathryn M. Lewis, Pak C. Sham, Robin M. Murray, Evangelos Vassos, Marta Di Forti

**Affiliations:** 1 Social, Genetic and Developmental Psychiatry Centre, Institute of Psychiatry, Psychology and Neuroscience, King's College London, London SE5 8AF, UK; 2 South London and Maudsley NHS Mental Health Foundation Trust, London, UK; 3 Department of Psychosis Studies, Institute of Psychiatry, King's College London, De Crespigny Park, Denmark Hill, London SE5 8AF, UK; 4 Genetics and Genomic Sciences, Icahn School of Medicine at Mount Sinai NYC, New York, NY, USA; 5 Department of Psychiatry, Washington University School of Medicine, St Louis, MO, USA; 6 Division of Psychological Medicine and Clinical Neurosciences, MRC Centre for Neuropsychiatric Genetics and Genomics, Cardiff University, Cardiff CF24 4HQ, UK; 7 Addiction and Mental Health Group (AIM), Department of Psychology, University of Bath, Bath, UK; 8 Department of Psychiatry and Neuropsychology, School for Mental Health and Neuroscience, South Limburg Mental Health Research and Teaching Network, Maastricht University Medical Centre, P.O. Box 616, 6200 MD Maastricht, The Netherlands; 9 Department of Experimental Biomedicine and Clinical Neuroscience, University of Palermo, Via G. La Loggia 1, 90129 Palermo, Italy; 10 ESRC Centre for Society and Mental Health and Health Service and Population Research Department, Institute of Psychiatry, King's College London, De Crespigny Park, Denmark Hill, London SE5 8AF, UK; 11 Department of Psychiatry, University of Cambridge, Herchel Smith Building for Brain & Mind Sciences, Forvie Site, Robinson Way, Cambridge CB2 0SZ, UK; 12 Rivierduinen Institute for Mental Health Care, Sandifortdreef 19, 2333 ZZ Leiden, The Netherlands; 13 Section of Psychiatry, Azienda Ospedaliera Universitaria Integrata di Verona, Piazzale L.A. Scuro 10, 37134 Verona, Italy; 14 Department of Medical and Surgical Science, Psychiatry Unit, Alma Mater Studiorum Università di Bologna, Viale Pepoli 5, 40126 Bologna, Italy; 15 INSERM, U955, Equipe 15, 51 Avenue de Maréchal de Lattre de Tassigny, 94010 Créteil, France; 16 Department of Child and Adolescent Psychiatry, Institute of Psychiatry and Mental Health, Hospital General Universitario Gregorio Marañón, IiSGM, CIBERSAM, School of Medicine, Universidad Complutense, Madrid, Spain; 17 Department of Medicine, Psychiatry Area, School of Medicine, Universidad de Oviedo, Centro de Investigación Biomédica en Red de Salud Mental (CIBERSAM), C/Julián Clavería s/n, 33006 Oviedo, Spain; 18 Department of Psychiatry, School of Medicine, Universidad de Valencia, CIBERSAM, Valencia, Spain; 19 Department of Psychiatry, Psychiatric Genetic Group, Instituto de Investigación Sanitaria de Santiago de Compostela, Complejo Hospitalario Universitario de Santiago de Compostela, Santiago de Compostela, Spain; 20 Department of Psychiatry, Mount Sinai School of Medicine, Icahn School of Medicine, New York, NY, USA; 21 Barcelona Clinic Schizophrenia Unit, Neuroscience Institute, Hospital Clinic, Department of Medicine, University of Barcelona, IDIBAPS, CIBERSAM, Barcelona, Spain; 22 Division of Psychiatry, Department of Neuroscience and Behaviour, Ribeirão Preto Medical School, University of São Paulo, São Paulo, Brasil; 23 Departamento de Medicina Preventiva, Faculdade de Medicina, Universidade de São Paulo, Avenida Doutor Arnaldo 455, CEP 01246-903 São Paulo, Brasil; 24 Núcleo de Pesquina em Saúde Mental Populacional, Universidade de São Paulo, Avenida Doutor Arnaldo 455, CEP 01246-903 São Paulo, Brasil; 25 CAMEO Early Intervention Service, Cambridgeshire & Peterborough NHS Foundation Trust, Cambridge CB21 5EF, UK; 26 PsyLife Group, Division of Psychiatry, University College London, London, UK; 27 CMP B CHU, BP 69, 63003 Clermont Ferrand, Cedex 1, France; 28 Department of Psychiatry, Early Psychosis Section, Academic Medical Centre, University of Amsterdam, Meibergdreef 5, 1105 AZ Amsterdam, The Netherlands; 29 Department of Mental Health and Addiction Services, ASP Crotone, Crotone, Italy; 30 Univ Paris Est Creteil (UPEC), AP-HP, Hopitaux Universitaires ‘H. Mondor’, DMU IMPACT, INSERM, IMRB, Translational Neuropsychiatry, Fondation FondaMental, F-94010 Créteil, France; 31 Brain Centre Rudolf Magnus, Utrecht University Medical Centre, Utrecht, The Netherlands; 32 Department of Addiction, Institute of Psychiatry, King's College London, De Crespigny Park, Denmark Hill, London SE5 8AF, UK; 33 National Institute for Health Research (NIHR), Mental Health Biomedical Research Centre at South London and Maudsley NHS Foundation Trust and King's College London, London, UK; 34 Department of Psychiatry, The University of Hong Kong, Hong Kong, China; 35 Centre for Genomic Sciences, Li KaShing Faculty of Medicine, The University of Hong Kong, Hong Kong, China

**Keywords:** cannabis, first-episode psychosis, high-potency cannabis, polygenic risk score, psychosis, schizophrenia

## Abstract

**Background:**

The association between cannabis and psychosis is established, but the role of underlying genetics is unclear. We used data from the EU-GEI case-control study and UK Biobank to examine the independent and combined effect of heavy cannabis use and schizophrenia polygenic risk score (PRS) on risk for psychosis.

**Methods:**

Genome-wide association study summary statistics from the Psychiatric Genomics Consortium and the Genomic Psychiatry Cohort were used to calculate schizophrenia and cannabis use disorder (CUD) PRS for 1098 participants from the EU-GEI study and 143600 from the UK Biobank. Both datasets had information on cannabis use.

**Results:**

In both samples, schizophrenia PRS and cannabis use independently increased risk of psychosis. Schizophrenia PRS was not associated with patterns of cannabis use in the EU-GEI cases or controls or UK Biobank cases. It was associated with lifetime and daily cannabis use among UK Biobank participants without psychosis, but the effect was substantially reduced when CUD PRS was included in the model. In the EU-GEI sample, regular users of high-potency cannabis had the highest odds of being a case independently of schizophrenia PRS (OR daily use high-potency cannabis adjusted for PRS = 5.09, 95% CI 3.08–8.43, *p* = 3.21 × 10^−10^). We found no evidence of interaction between schizophrenia PRS and patterns of cannabis use.

**Conclusions:**

Regular use of high-potency cannabis remains a strong predictor of psychotic disorder independently of schizophrenia PRS, which does not seem to be associated with heavy cannabis use. These are important findings at a time of increasing use and potency of cannabis worldwide.

## Introduction

Cannabis is used by over 200 million people worldwide, and the prevalence of use has increased in many countries in recent years, as has the potency of the cannabis available and the number of people seeking treatment for cannabis-related problems (European Monitoring Centre for Drugs and Drug and Addiction, 2016; Freeman et al., 2019; Grucza, Agrawal, Krauss, Cavazos-Rehg, & Bierut, 2016; Manthey, 2019). Prospective epidemiological and biological studies (Gage, Hickman, & Zammit, 2016; Murray et al., 2017) suggest a causal link between cannabis use and psychotic disorder, and evidence supports a dose–response association (Di Forti et al., 2019; Marconi, Di Forti, Lewis, Murray, & Vassos, 2016). Frequent cannabis use and use of high-potency types have been linked to variations in the incidence of psychotic disorder across Europe (Di Forti et al., 2019; Gonçalves-Pinho, Bragança, & Freitas, 2020; Hjorthøj, Posselt, & Nordentoft, 2021; Rognli et al., 2023), North America (Callaghan et al., 2022; Moran, Tsang, Ongur, Hsu, & Choi, 2022), and in the Global South (Lee Pow et al., 2023). These findings have raised the important issue of whether increased consumption particularly of high-potency cannabis will lead to an increase in the incidence of psychosis (Murray & Hall, 2020).

Patterns of cannabis use such as lifetime cannabis use (never/ever used) and cannabis use disorder (CUD) are influenced by genetic factors (Agrawal & Lynskey, 2006; Pasman et al., 2018). Heritability from twin studies is approximately 45% for lifetime cannabis use and between 51% and 70% for CUD (Kendler et al., 2015; Verweij et al., 2010). Narrow-sense heritability, based on estimates from single-nucleotide polymorphisms (SNPs) only, is estimated at 11% for lifetime cannabis use and 12% for CUD (Johnson et al., 2020; Pasman et al., 2018). Genome-wide association studies (GWASs) have also shown a significant genetic correlation between lifetime cannabis use or CUD and schizophrenia (Demontis et al., 2019). Moreover, polygenic risk scores (PRSs) for schizophrenia have been reported to explain a small but significant proportion of the variance in lifetime cannabis use, quantity of cannabis used (Power et al., 2014), and CUD (Demontis et al., 2019). Previous studies have shown that individuals at high risk for psychotic disorder (Vadhan, Corcoran, Bedi, Keilp, & Haney, 2017) and/or with a known family for psychosis (Henquet, Murray, Linszen, & van Os, 2005), are more vulnerable to the psychotogenic effect of cannabis use (Verweij et al., 2017).

We have used data on patterns of cannabis use (frequency of use and, where available, potency of the type used) along with genotype data from two studies: the European Network of National Schizophrenia Networks Studying Gene–Environment Interactions (EU-GEI) case-control study and the UK Biobank. We used both datasets to investigate the following questions: (1) is schizophrenia liability, as measured by the PRS, associated with lifetime cannabis use and/or patterns of cannabis use in population controls and in subjects with a diagnosis of psychotic disorder? (2) What are the independent and combined effects of schizophrenia PRS and cannabis use on odds of psychotic disorder? (3) To what extent does adding schizophrenia PRS data to information on patterns of cannabis use improve the identification of those heavy cannabis users who will develop psychotic disorder?

## Methods

### Samples

We analyzed data from two independent studies: first, we used a first-episode psychosis case-control sample, the EU-GEI, a multi-center case-control study of the genetic and environmental determinants of psychotic disorders. First-episode psychosis patients (FEPp) and population-based controls were recruited between May 2010 and April 2015 in 17 catchment areas in England, France, the Netherlands, Italy, Spain, and Brazil (Jongsma et al., 2018). Ethical approval was provided by relevant research ethics committees in each of the study sites.

Second, we conducted a comparative analysis using data from UK Biobank, a population-based study, including over 500 000 UK-based participants. The UK Biobank study was approved by the North-West Research Ethics Committee (ref 06/MREC08/65) in accordance with the Helsinki Declaration of 1975. All participants of both UK Biobank and EU-GEI provided written informed consent.

### Participants

#### EU-GEI study

FEPp were included if (a) aged 18–64 years and (b) resident within the study areas at the time of their first presentation, and received a diagnosis of psychosis (ICD-10 F20-29); further details are provided in the online Supplementary methods and previous publications (Di Forti et al., 2019; Quattrone et al., 2021). All cases interviewed received a research-based diagnosis (McGuffin, Farmer, & Harvey, 1991; Quattrone et al., 2019). FEPp were excluded if (a) previously treated for psychosis, (b) they met criteria for organic psychosis (ICD-10: F09), or for a diagnosis of transient psychotic symptoms resulting from acute intoxication (ICD-10: F1X.5). Controls were excluded if they had received a diagnosis of, and/or treatment for, psychotic disorder.

#### UK Biobank

Subjects aged 40–70 years were recruited from 22 UK assessment centers. This study has been described in detail previously, see Bycroft et al. (2018) and Bycroft et al. (2018). All necessary demographic, medical, and genetic data were downloaded from UK Biobank. Cases were defined as any participant with either a recorded diagnosis of psychotic disorder, identified through recorded ICD-10 data (codes F20–F29), or who self-reported ‘schizophrenia’ or ‘Any other type of psychosis or psychotic illness’ as part of the online Mental Health Questionnaire (MHQ) (in response to the question: ‘Have you been diagnosed with one or more of the following mental health problems by a professional, even if you don't have it currently?’). Participants without psychosis were defined as any UK Biobank participant who had no reported psychotic disorder or previous treatment with an antipsychotic. We compared the baseline demographic data as well as information on the prescription of antipsychotics to consider the differences between cases defined by ICD-10 criteria and through self-report (see online Supplementary materials).

### Sociodemographic variables

#### EU-GEI study

Data on age and sex were collected using the Medical Research Council Sociodemographic Schedule modified version (Mallett, Leff, Bhugra, Pang, & Zhao, 2002).

#### UK Biobank

Information on age and sex was collected at recruitment when participants provided sociodemographic details (Bycroft et al., 2018).

### Measures of cannabis use

Data on patterns of cannabis use were collected from the EU-GEI study using the modified Cannabis Experience Questionnaire further updated (CEQ_EU-GEI_) (Di Forti et al., 2019). The following measures of cannabis use were recorded: (1) age at first use of cannabis; (2) lifetime frequency of use, and (3) the potency of the cannabis used (Di Forti et al., 2015). Potency was estimated as described in Di Forti et al. (2019) using published data on the types of cannabis available and its potency from each of the sites included in this paper (see online Supplementary materials for a detailed discussion on this variable) (Brisacier et al., 2015; de Oliveira, Voloch, Sztulman, Neto, & Yonamine, 2008; European Monitoring Centre for Drugs and Drug and Addiction, 2016; Niesink, Rigter, Koeter, & Brunt, 2015; Potter, Clark, & Brown, 2008; Potter, Hammond, Tuffnell, Walker, & Di Forti, 2018; Zamengo, Frison, Bettin, & Sciarrone, 2014). We used the lifetime frequency of use and the cannabis potency variables to build the ‘*frequency-type* composite cannabis use measure’ that we previously found (Di Forti et al., 2015) and replicated (Murray et al., 2017) to be a strong predictor of psychotic disorder independently of other drugs of abuse, age, gender, ethnicity, site, and level of education. Study participants reported in their language the name of the type of cannabis used. Low-potency cannabis was defined as cannabis with a tetrahydrocannabinol (THC) concentration of less than 10% (THC < 10%), and high-potency cannabis was defined as THC concentration as greater or equal to 10% (THC ⩾ 10%).

In the UK Biobank sample, three specific questions on cannabis use were recorded as part of the online MHQ. We used these data to identify those subjects that had (a) never used cannabis, (b) used cannabis at least once, (c) used cannabis weekly at some stage, and (d) used cannabis daily at some stage. Data on potency or age of first use were not captured.

### Choice of primary outcome measure

The primary outcome measure is defined as case status. In both cohorts, cases were included if they had a diagnosis of psychosis defined as ICD-10 codes F20–29. In the UK Biobank replications sample, we also included participants who self-reported a psychosis diagnosis. A comparison between those cases with a defined ICD-10 diagnosis and self-report only is provided in the online Supplementary materials. The measures of cannabis use as described above were chosen based on available data in our two cohorts.

### Genotyping

Genotyping and imputation of EU-GEI and UK Biobank subjects has been described previously (Bycroft et al., 2018; Quattrone et al., 2021). Briefly, EU-GEI samples were genotyped at the MRC Centre for Neuropsychiatric Genetics and Genomics in Cardiff (UK) using a custom Illumina HumanCoreExome-24 BeadChip genotyping array covering 570 038 genetic variants (Quattrone et al., 2021). Genotyping for UK Biobank participants was undertaken using the Affymetrix UK BiLEVE Axiom array (used for the first ~50 000 participants) and the Affymetrix UK Biobank Axiom Array (~450 000 participants) (Bycroft et al., 2018). The Haplotype Reference Consortium (The Haplotype Reference Consortium, 2016) and the UK10K consortium (Huang et al., 2015; UK10K Consortium, 2015) were used as imputation panels. Relatedness between participants was assessed using kinship scores provided by UK Biobank. One of each related pair (KING *r*^2^ > 0.044) (Manichaikul et al., 2010) was removed using the GreedyRelated algorithm, which prioritizes including cases (Choi, 2020). For both samples, we calculated genetic principal components to assess population stratification and used these to assign genetic ancestry using the Genopred pipeline, using 1000 Genomes data as the reference populations (1000 Genomes Project Consortium, 2010; Pain; Pain et al., 2021). Participants who were not assigned to an ancestry group were excluded from our analyses (see online Supplementary materials).

### PRS calculation

PRSs were calculated separately for different ancestry groups to account for population-specific differences in linkage disequilibrium and allele frequency. PRSs for schizophrenia were generated for participants of European ancestry (EUR) and East Asian ancestry (EAS) using PRS-CS (Ge, Chen, Ni, Feng, & Smoller, 2019) and Plink (Purcell et al., 2007), based on GWAS summary statistics from the Schizophrenia Working Group of the Psychiatric Genomics Consortium (PGC) wave three (with EU-GEI samples excluded) (Trubetskoy et al., 2022). For individuals of African ancestry PRSs were generated using PRS-CSx (Lee, Goddard, Wray, & Visscher, 2012; Lewis & Vassos, 2017), which works in the same way as PRS-CS but allows for inclusion of multiple sets of summary statistics from different populations to improve predictive power. To calculate the AFR PRS, we used the EUR summary statistics described previously in combination with summary statistics from the Genomic Psychiatry Cohort (Bigdeli et al., 2020). PRS for CUD were calculated using EUR and AFR GWAS summary statistics (Levey et al., 2023) using PRS-CS and PRS-CSx respectively, as described above. Once calculated, these ancestry-specific scores were combined into a single column and all groups analyzed together. Due to the large dominance of the EUR ancestry participants in both cohorts, we were unable to perform population-specific analyses in the EAS or AFR group. Each PRS was standardized to mean of 0 and standard deviation of 1 (Lewis & Vassos, 2017).

### Statistical analysis

Adjusted logistic regression models were run to estimate: (1) odds of cannabis use for each unit increase in schizophrenia PRS and (2) the independent and combined effect of the selected measures of cannabis use and the schizophrenia PRS on the odds ratio (OR) for psychotic disorder. We fitted multiplicative interaction terms to the logistic models to test if schizophrenia PRS modified the effect of cannabis use on the OR for psychotic disorder. Interaction analyses were conducted in EUR participants only, due to limited numbers of non-EUR participants. All regression models were adjusted for: the first 10 principal components, recruitment site, age, sex, and tobacco smoking (as defined in our previous publication; Di Forti et al., 2019). The latter was added due to the clinical and genetic overlap among schizophrenia, tobacco use, and cannabis use (Johnson et al., 2020). We conducted additional analyses adjusting for CUD PRS, to consider the impact of underlying genetic risk for CUD on patterns of cannabis use and schizophrenia case status. For each model performed, we calculated Nagelkerke's *R*^2^ to consider model fit and converted this observed scale *R*^2^ measure to a liability scale measure (Lee et al., 2012). We used 0.1 as an estimate for the population level lifetime risk for psychosis. To mitigate potential statistical confounding in the interaction models, we carried out additional analyses adding covariate × environment and covariate × gene interaction terms to the model, as recommended by previous publication (see online Supplementary materials section) (Keller, 2014). We calculated the positive-predictive value (PPV) for both the schizophrenia and CUD PRS, using the pROC package in R (Robin et al., 2011). All analyses were conducted using R version 4.1.1 (R Core Team., 2021).

## Results

### Baseline characteristics of study participants

#### EU-GEI

In total, 1130 FEPp and 1497 population controls consented to take part. The total sample with available genetic data and data on cannabis use was 945 controls and 647 cases (total 1592). We defined the following ancestry groups to build the schizophrenia PRS by ancestry: *N*_EUR_ = 1262, *N*_SAS_ = 35, *N*_AFR_ = 192, *N*_EAS_ = 18, *N*-undefined = 409; the latter were excluded. All analyses reported here are the results of the full sample. Given the differences in predictive power of polygenic scores across ancestry groups, we report a EUR-only sensitivity analysis in the online Supplementary materials.

The final EU-GEI sample consisted of 405 FEPp (cases) and 693 controls (see recruitment flow chart in the online Supplementary materials). As highlighted in Table 1, cases were younger and more likely to be men than controls. Cases were also more likely to have tried cannabis, to have first used it at age 15 years old or younger, and to have used it daily. Cases were also more likely to have used more potent types and to have used them daily than controls.

**Table 1.** Differences between cases and controls in across sociodemographic factors and patterns of cannabis use

		EU-GEI					UK Biobank				
		Controls	Case^a^	df	Test stat	*p*	Controls	Case^b,c^	df	Test stat	*p*
		(*N* = 693)	(*N* = 405)				(*N* = 142 857)	(*N* = 743)			
Age		38.06 (13.30)	32.05 (11.13)	966.8	*t* = 8.03	2.9 × 10^−15^	56.03 (7.71)	54.66 (8.12)	770.29	*t* = 4.66	3.76 × 10^−6^
Mean (s.d.)											
Sex		323 (46.54)	246 (60.74)	1	χ^2^ = 20.09	7.4 × 10^−6^	62 235 (43.56)	325 (43.74)	1	χ^2^ = 0.01	0.92
*N* male (% male)											
Genetic ancestry *N* (%)	AFR	11 (57.89)	8 (42.11)	2	χ^2^ = 0.8	0.7	139 132 (99.47)	735 (0.53)	2	χ^2^ = 23	1 × 10^−5^
	EAS	23 (48.94)	24 (51.06)				1044 (99.43)	6 (0.57)			
	EUR	754 (62.68)	449 (37.32)				1148 (98.46)	18 (1.54)			
Lifetime cannabis use		355 (51.15)	275 (67.90)	2	χ^2^ = 48.69	2.7 × 10^−11^	30 855 (21.60)	254 (34.19)	1	χ^2^ = 68.46	<2.2 × 10^−16^
*N* used (% used)											
Age at first use ⩽15		90 (12.99)	114 (28.15)	2	χ^2^ = 79.74	2.7 × 10^−11^	N/A				
*N* ⩽ 15 (% ⩽15)^d^											
Frequency of use^e^ *N* (%)	Never used	339 (48.92)	130 (32.10)	4	χ^2^ = 79.13	2.7 × 10^−16^	112 002 (78.40)	489 (65.81)	5	χ^2^ = 72.07	3.8 × 10^−14^
	Rare use	136 (19.62)	55 (13.58)				19 601 (13.72)	126 (16.96)			
	<1/week	127 (18.33)	60 (14.81)				3557 (2.49)	21 (2.83)			
	Weekly	51 (7.36)	45 (11.11)				4839 (3.39)	55 (7.40)			
	Daily	40 (5.77)	107 (26.42)				1928 (1.35)	46 (6.19)			
Potency of cannabis used^f^^,^^g^ *N* (%)	Never used	339 (48.92)	130 (32.10)	2	χ^2^ = 42.67	5.4 × 10^−10^	N/A				
	THC < 10%	180 (25.97)	90 (22.22)								
	THC ⩾ 10%	151 (21.79)	154 (38.02)								
Type-frequency composite cannabis use measure^f^^,^^h^ *N* (%)	Never used	339 (48.92)	130 (32.10)	6	χ^2^ = 121.42	<2.2 × 10^−16^	N/A				
	Rare use of THC < 10%	133 (19.19)	41 (10.12)								
	Rare use of THC ⩾ 10%	107 (15.44)	51 (12.59)								
	Weekly use of THC < 10%	29 (4.18)	21 (5.19)								
	Weekly use of THC ⩾ 10%	21 (3.03)	24 (5.93)								
	Daily use of THC < 10%	18 (2.60)	27 (6.67)								
	Daily use of THC ⩾ 10%	22 (3.17)	78 (19.26)								

df, degrees of freedom; s.d., standard deviation; THC, tetrahydrocannabinol.

a Cases for EU-GEI study defined as first-episode psychosis patients; cases for UK Biobank defined as either ^b^Schizophrenia or psychosis based on self-report and/or ICD-10 code or ^c^any major psychiatric disorder defined by ICD-10 code.

d EU-GEI study recorded data on age at first use, UK Biobank recorded data on age at last use.

e In EU-GEI data, eight cases (1.98%) did not provide this information. In UK Biobank data 864 (0.63%) controls and six (0.81%) cases did not provide this information.

f Data on potency not recorded for UK Biobank study.

g 23 controls (3.32%) and 31 (7.65%) cases did not provide this data.

h 24 controls (3.46%) and 33 (8.15%) cases did not provide this data.

#### UK Biobank

Data for a total of 455 538 UK Biobank participants with high-quality genetic data were downloaded. Of these, approximately 32% also had responded to the MHQ and thus provided data on previous cannabis use, giving us a final working sample of 145 244 (*N*_EUR_ = 143 600, *N*_AFR_ = 1177, *N*_EAS_ = 527). This final sample consisted of 743 psychosis cases, as defined by a combination of ICD-10 data and self-report, and 142 857 participants without psychosis (additional detail on case ascertainment for the UK Biobank replication study provided in the online Supplementary materials).

#### PRS distribution

**Figure 1.**
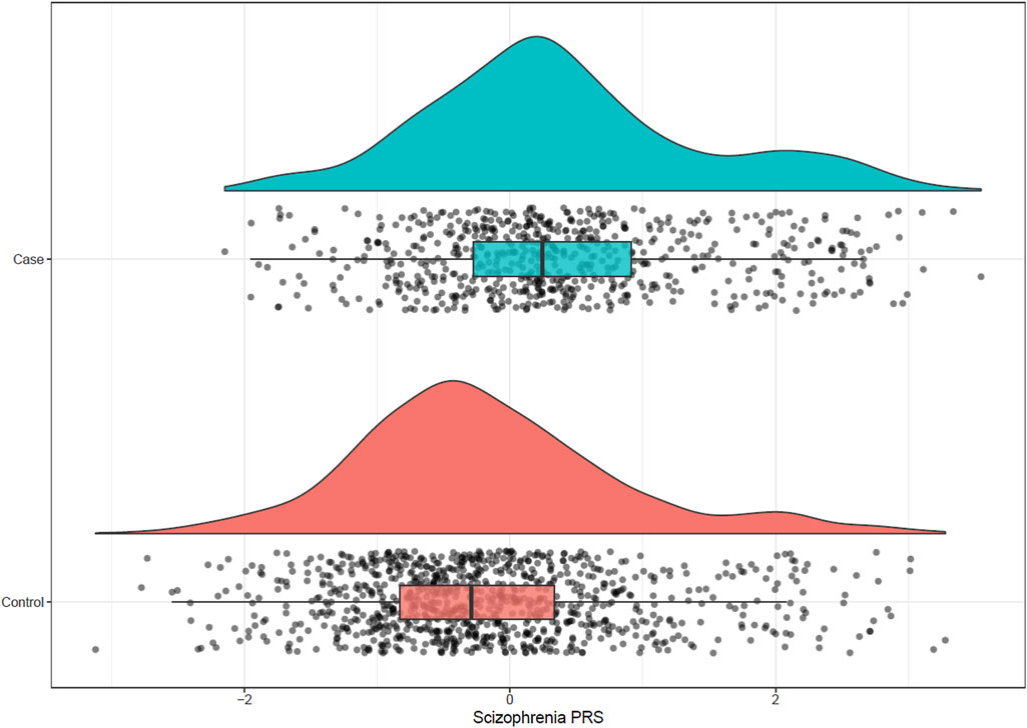
Distribution of schizophrenia PRSs shows an overall increase in PRS for cases compared to controls in the EU-GEI cohort (mean PRS cases = 0.40 ± 1.05, mean PRS controls = 0.17 ± 1.00, *p* = 2.2 × 10^−26^).

The schizophrenia PRS was on average higher in FEPp than in controls (Fig. 1): EU-GEI case mean schizophrenia PRS = 0.40, s.d. = 1.05; controls mean schizophrenia PRS = −0.17, s.d. = 1.00; *t* = −10.86, df = 1349.3; *p* = 2.2 × 10^−26^. There were more controls in the schizophrenia PRS quintile 1 compared to cases, while the opposite was true in quintile 5: controls quintile 1 = 251/319 (78.68%); cases quintile 1 = 68/319 (21.32%); controls quintile 5 = 136/318 (42.77%); cases quintile 5 = 182/318 (57.23%) (χ^2^ = 127.33, df = 4, *p* = 1.45 × 10^−26^). The CUD PRS was also higher in EU-GEI first-episode psychosis cases compared to controls: EU-GEI case mean PRS = 0.31 ± 1.1, controls mean CUD PRS = −0.1 ± 0.98, *p* diff = 7.63 × 10^−14^). We observed similar patterns in UK Biobank data (see online Supplementary materials).

#### Variance explained and PPV of schizophrenia and CUD PRS

We calculated pseudo-*R*^2^ statistics (Nagelkerke and liability scale, adjusted for the sample prevalence) by each of our predictors. In the EU-GEI cohort, a model including schizophrenia PRS, site, sex, and 10 principal components explained 25.9% of the variation in case-control status (12.8% on the liability scale). When tobacco smoking (more or less than 10 cigarettes per day) was included, the variance explained was 34.2% on the observed and 18.0% on the liability scale. Adding daily cannabis use increased this to 50.3% on the observed and 29.8% on the liability scale. This was not significantly increased by adding age at first use (*R*^2^_obs_ = 50.3%, *R*^2^_liab_ = 30.1%) but was increased by adding the use of high-potency cannabis (THC > 10%) (*R*^2^_obs_ = 55.5%, *R*^2^_liab_ = 34.7%). Adding the CUD PRS did not improve the model (*R*^2^_obs_ = 56.0%, *R*^2^_liab_ = 34.7%) (online Supplementary S-Fig. 10).

In the UKB cohort, a model including schizophrenia PRS, site, sex, age, and 10 principal components explained 1.3% (2.2% on the liability scale). When tobacco smoking (more or less than 10 cigarettes per day) was included, the variance explained was 1.7% on the observed and 2.9% on the liability scale. Adding daily cannabis use increased this to 29.3% on the observed and 48.3% on the liability scale (online Supplementary S-Fig. 11). CUD PRS was not associated with schizophrenia status and did not increase the variance explained 29.3% (48.3% on the liability scale).

In our EU-GEI control sample alone, schizophrenia PRS and 10 principal components explained a small but non-significant proportion of the variance between those who never used cannabis and (a) those who had tried at least once (lifetime use *R*^2^ = 7.86%; *p* schizophrenia PRS = 0.08), (b) those who had started at age 15 or younger (*R*^2^ = 3.89%; *p* schizophrenia PRS = 0.3), (c) having used it daily (*R*^2^ = 3.14%; *p* schizophrenia PRS = 0.21), and (d) using high-potency types (*R*^2^ = 18.38%; *p* = 0.41). In our EU-GEI control sample alone, CUD PRS and 10 principal components explained a significant proportion of the variance between those who never used cannabis and (a) having tried it at least once (lifetime use *R*^2^ = 8.31%; *p* schizophrenia PRS = 0.03), and (b) having used it daily (*R*^2^ = 4.52%; *p* schizophrenia PRS = 0.01) but was not associated with use of high-potency types (*R*^2^ = 18.56%; *p* = 0.62) or having started at age 15 or younger (*R*^2^ = 4.08%; *p* schizophrenia PRS = 0.96).

In the EU-GEI cohort, we calculated the PPV for assigning psychosis case/control status to be 0.65 for schizophrenia PRS and 0.63 for CUD PRS.

#### Does schizophrenia PRS predict cannabis initiation and/or patterns of cannabis use?

Regression adjusted for age, sex, tobacco smoking, recruitment site, and for the 10 principal components showed that schizophrenia PRS was not associated with cannabis initiation (lifetime cannabis use yes/no) among cases or controls from EU-GEI (EU-GEI cases: OR = 0.9; 95% confidence interval [CI] 0.63–1.3; *p* = 0.58; EU-GEI controls: OR = 1.14; 95% CI 0.9–1.44; *p* = 0.28). In addition, schizophrenia PRS did not explain how frequently either cases or controls used cannabis, including when we specifically compared never use with daily use: (EU-GEI cases: OR = 0.78; 95% CI 0.53–1.15; *p* = 0.22; EU-GEI controls: OR = 1.25; 95% CI 0.82–1.94; *p* = 0.31). Schizophrenia PRS also did not predict age at first use among either cases or controls: (EU-GEI cases: *β* = 0.08; s.e. = 0.36, *p* = 0.83; EU-GEI controls: *β* = −0.24; s.e. = 0.34, *p* = 0.48) (Table 2).

**Table 2.** ORs for varying measures of cannabis use in EU-GEI and UK Biobank cohorts

	EU-GEI case				EU-GEI controls			
	*N*	OR	95% CI	*p*	*N*	OR	95% CI	*p*
Scz. PRS predicting lifetime use	615	0.9	0.63–1.30	0.58	926	1.14	0.90–1.44	0.28
Scz. PRS predicting weekly use	439	1.21	0.75–1.98	0.44	858	1.18	0.73–1.94	0.52
Scz. PRS predicting daily use	527	0.78	0.53–1.15	0.22	869	1.25	0.82–1.94	0.31
Scz. PRS predicting age at first use	407	0.08	−0.63–0.78	0.83	421	−0.24	−0.91–0.43	0.48
Scz. PRS predicting potency of use	419	1.08	0.71–1.66	0.72	716	1.15	0.89–1.51	0.29
	UKBB case				UKBB controls			
	*N*	OR	95% CI	*p*	*N*	OR	95% CI	*p*
Scz. PRS predicting lifetime use	759	0.94	0.76–1.16	0.57	141 326	1.08	1.07–1.10	9.72 × 10^−23^
Scz. PRS predicting weekly use	706	0.87	0.59–1.31	0.50	138 464	1.09	1.06–1.13	6.95 × 10^−7^
Scz. PRS predicting daily use	547	1.05	0.64–1.77	0.85	112 693	1.14	1.07–1.20	1.61 × 10^−5^

All models compared to never users as reference group, and adjusted for age, sex, recruitment site, tobacco smoking, and 10 principal components.

Regression adjusted for age, sex, tobacco smoking, recruitment site, and the 10 principal components showed that CUD PRS did predict cannabis initiation (lifetime cannabis use yes/no) among cases or controls from EU-GEI (EU-GEI cases: OR = 1.57; 95% CI 1.03–2.4; *p* = 0.03; EU-GEI controls: OR = 1.36; 95% CI 1–1.86; *p* = 0.05). In addition, CUD PRS was significantly associated with weekly use among controls only, and daily use among cases only: EU-GEI cases OR (weekly use) = 2.22; 95% CI 1.31–3.85; *p* = 0.004; EU-GEI controls OR (weekly use) = 0.91; 95% CI 0.49–1.68; *p* = 0.76; EU-GEI cases OR (daily use) = 1.26; 95% CI 0.8–1.98; *p* = 0.31; EU-GEI controls OR (daily use) = 1.84; 95% CI 1.05–3.28; *p* = 0.04. CUD PRS did not predict age at first use among cases or controls: (EU-GEI cases: *β* = 0; s.e. = 0.42, *p* = 1; EU-GEI controls: *β* = −0.12; s.e. = 0.43, *p* = 0.77) (online Supplementary S-Tables 12, 13, S-Fig. 13).

We saw similar patterns among UK Biobank cases. However, we found an association of small magnitude between schizophrenia PRS and both lifetime cannabis use and frequency of use in participants without psychosis (UK Biobank participants without psychosis lifetime use OR = 1.08; 95% CI 1.07–1.09; *p* = 9.72 × 10^−23^; UK Biobank participants without psychosis weekly use OR = 1.09, 95% CI 1.06–1.13 *p* = 6.95 × 10^−7^; UK Biobank participants without psychosis daily users OR = 1.14, 95% CI 1.07–1.20, *p* = 1.61 × 10^−5^) (Table 2). Including the CUD PRS in these models led to a reduction in the magnitude of this effect (online Supplementary S-Tables 13, 14). For lifetime use, the effect size for schizophrenia PRS reduced by 39%, but the association with lifetime use remained significant. For daily use, the effect size for schizophrenia PRS reduced by 82% and was no longer significantly associated with lifetime cannabis use. By comparison, a model including only CUD PRS was less impacted by the addition of schizophrenia PRS, with a reduction of effect size for CUD PRS of 23 and 3% for lifetime and daily use respectively (online Supplementary S-Tables 14, 15, S-Fig. 14).

#### The independent and combined effect of schizophrenia PRS and pattern of cannabis use on the OR for psychotic disorder

In the EU-GEI sample, both adjusted and unadjusted regression for schizophrenia PRS showed that lifetime cannabis use was associated with an increased risk for psychotic disorder (adjusted OR (inc. schizophrenia PRS) = 1.63; 95% CI 1.26–2.12; *p* = 2.32 × 10^−4^; unadjusted OR = 1.67; 95% CI 1.3–2.15; *p* = 6.53 × 10^−5^). Weekly cannabis use was also significantly associated with increased odds of psychosis (adjusted OR = 2.31; 95% CI 1.52–3.51; *p* = 8.72 × 10^−5^; unadjusted OR = 2.42; 95% CI 1.62–3.63; *p* = 1.67 × 10^−5^), and the strongest association was with daily cannabis (OR = 3.7; 95% CI 2.59–5.35; *p* = 1.53 × 10^−12^; unadjusted OR = 3.7; 95% CI 2.62–5.28; *p* = 2.61 × 10^−12^) (Table 3). In models additionally adjusted for CUD PRS, we demonstrate that CUD PRS is also associated with case-control status in the EU-GEI cohort, with little evidence that the CUD PRS confounds the results for the schizophrenia PRS (difference in schizophrenia PRS OR for models with and without CUD PRS <10% in all cases) (online Supplementary S-Table 17, S-Fig. 15). These results were replicated in the UK Biobank sample (Table 3, although in all analyses the CUD PRS was not associated with psychosis status) (online Supplementary S-Table 18). We fitted interaction terms to the logistic models to consider the putatively modifying effect of schizophrenia PRS on the impact of cannabis use on the OR for psychotic disorder and found no evidence of an interaction (Table 3, online Supplementary S-Table 9; for additional discussion of interaction models in both cohorts see online Supplementary materials and S-Tables 10, 11).

**Table 3.** Independent and combined effects of schizophrenia PRS and cannabis use measures on risk for psychosis in the EU-GEI and UK Biobank

	EU-GEI				UK Biobank			
Predictors	*N*	OR	95% CI	*p*	*N*	OR	95% CI	*p*
**Panel A**								
Scz. PRS	1541	2.85	2.31–3.54	5.04 × 10^−22^	142 085	1.29	1.18–1.40	1.99 × 10^−8^
Lifetime cannabis use		1.63	1.26–2.12	2.32 × 10^−4^		1.51	1.27–1.78	1.53 × 10^−6^
Scz. PRS	1297	2.94	2.34–3.72	1.29 × 10^−16^	139 170	1.28	1.17–1.40	1.06 × 10^−7^
Weekly cannabis use		2.31	1.52–3.51	8.72 × 10^−5^		2.01	1.50–2.64	1.07 × 10^−6^
Scz. PRS	1396	2.80	2.24–3.53	6.43 × 10^−19^	113 240	1.29	1.16–1.43	1.59 × 10^−6^
Daily cannabis use		3.70	2.59–5.35	1.53 × 10^−12^		4.17	2.93–5.82	2.63 × 10^−16^
**Panel B**								
Scz. PRS	1541	2.73	2.13–3.51	3.62 × 10^−15^	142 085	1.28	1.16–1.42	1.72 × 10^−6^
Lifetime cannabis use		1.61	1.23–2.10	4.55 × 10^−4^		1.51	1.27–1.78	2.18 × 10^−6^
Schizophrenia PRS × lifetime use		1.08	0.85–1.38	0.571		1.00	0.86–1.18	0.974
Scz. PRS	1297	2.92	2.32–3.72	5.17 × 10^−19^	139 170	1.31	1.19–1.44	2.24 × 10^−8^
Weekly cannabis use		2.29	1.50–3.50	1.15 × 10^−4^		2.06	1.54–2.71	4.48 × 10^−7^
Schizophrenia PRS × weekly use		1.05	0.70–1.64	0.809		0.79	0.62–1.02	0.055
Scz. PRS	1396	2.89	2.30–3.66	3.70 × 10^−19^	113 240	1.28	1.15–1.42	6.12 × 10^−6^
Daily cannabis use		3.87	2.69–5.60	4.45 × 10^−13^		4.08	2.84–5.74	4.10 × 10^−15^
Schizophrenia PRS × daily use		0.79	0.57–1.11	0.169		1.10	0.82–1.55	0.535

Panel A illustrates the main effect of schizophrenia PRS and frequency of cannabis use, independent of each other, on the risk of psychosis. Panel B illustrates the results for models including schizophrenia PRS and frequency of cannabis use in interaction. All models adjusted for age, sex, recruitment site, tobacco smoking, and 10 principal components.

**Figure 2.**
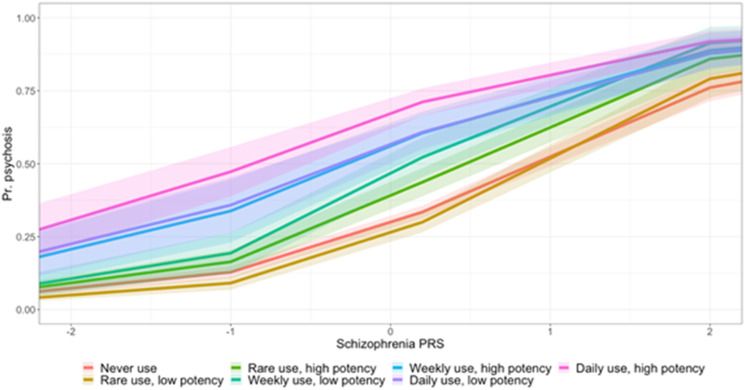
Probability of psychosis case status within EU-GEI cohort as schizophrenia PRS increases, across seven levels of cannabis potency–frequency composite measure.

We observed that those who used either high- or low-potency cannabis on a daily basis had an increase in the risk for psychotic disorder compared to never users, independently of their schizophrenia PRS and after adjusting for age, sex, site, and 10 principal components, with the greatest magnitude of effect observed in high-potency daily users (low-potency daily OR = 3.02; 95% CI 1.80–5.08; *p* = 3.13 × 10^−5^; high-potency daily OR = 5.09; 95% CI 3.08–8.43; *p* = 3.21 × 10^−10^) (online Supplementary S-Tables 5, 6). We fitted interaction terms to the logistic models and observed no evidence of a modifying effect of schizophrenia PRS and cannabis use on the OR for psychotic disorder, although there did appear to be a trend increase in psychosis risk across all levels of use (Fig. 2).

## Discussion

This study is the first to provide estimates of risk for psychotic disorder by the joint modeling of cannabis use (frequency) and common variant liability to schizophrenia. In keeping with previous studies (Di Forti et al., 2015, . 2019; Marconi et al., 2016), our analyses reveal an association between case-control status in both cohorts. Lifetime cannabis use was associated with increased odds of psychosis, and the magnitude of this effect was greater when considering those users who consumed cannabis more regularly (weekly and daily use). These results remained consistent when we adjusted for the schizophrenia PRS, demonstrating that genetic risk is independent of the environmental risk factor that is cannabis use. In the EU-GEI sample, we also see independent effects of high-potency cannabis use when adjusting for schizophrenia PRS. When we fitted interaction terms to these models, we found little evidence for a modifying effect of schizophrenia PRS.

In the EU-GEI cohort, we found that schizophrenia PRS was not associated with an individual's propensity to try cannabis or, among users, with the frequency, or other patterns of use. These findings are consistent with a recent cross-sectional study of patients with established psychosis (using a different EU-GEI cohort of chronic schizophrenia patients), which showed no evidence of correlation between schizophrenia PRS and regular cannabis use (Guloksuz et al., 2019).

Among the UK Biobank sample of participants without psychosis we found that schizophrenia PRS was associated with patterns of cannabis use (explaining less than 1% of the variance in lifetime cannabis use and daily use). When we included the CUD PRS in these models the effect size for schizophrenia PRS was reduced by 39% and 82% for lifetime and daily use, respectively, indicative of substantial confounding by the CUD PRS. We also observed evidence of confounding by the schizophrenia PRS on the CUD PRS for lifetime use, but not daily use. Given more frequent cannabis use confers greater risk for psychosis, arguments of reverse causality (schizophrenia leading to heavy cannabis use) would be supported by evidence of an association between schizophrenia PRS and cannabis use patterns. Here, we show that while a higher genetic risk for schizophrenia may increase the chance of ever trying cannabis, it may have less bearing on the likelihood of becoming a heavy user. Nonetheless, there does seem to be some independent effects of schizophrenia genetic risk on cannabis use, which should be interrogated in other population-based cohorts.

Future research should implement robust causal inference methods to interrogate this reported association more thoroughly. Our study, which utilized an SNP-inclusive method to calculate polygenic scores, is not designed to confirm or exclude the possibility of a causal association. Previous studies investigating this relationship using genetic data have found conflicting results (Elkrief et al., 2023; Gillespie & Kendler, 2021; Hjorthoj et al., 2021; Jones et al., 2022; Verweij et al., 2017; Wainberg, Jacobs, di Forti, & Tripathy, 2021).

More recently, Mendelian randomization analyses have taken advantage of the available genetic data on both schizophrenia and cannabis use, but so far they have produced contradictory findings, perhaps due to variation in the cannabis use instrumental variables used (Cheng et al., 2023; Gage et al., 2017; Pasman et al., 2018; Vaucher et al., 2018). The recent well-powered GWAS on CUD found evidence of a bi-directional association between schizophrenia and CUD, with a larger magnitude of effect from CUD to schizophrenia (Levey et al., 2023).

In a commentary, Gillespie and Kendler (2021) discuss three possible scenarios, which might explain the complexity of the relationship between cannabis use and psychotic disorders. They point out that, for instance, cannabis use might be partly causal and partly confounded by genetic/familial effects and/or reverse causation, suggesting that ‘a causal role’ of cannabis use in psychotic disorders might not exclude a non-causal genetic confounding and vice versa. They indicate that more clarity might come from studies investigating changes in incidence rates of schizophrenia in places where rates of cannabis use have changed because of its legalization. These data are beginning to be published and further support a causal role of cannabis use in psychotic disorders (Callaghan et al., 2022; Gonçalves-Pinho et al., 2020; Hjorthøj et al., 2021).

Another important issue to consider is the relative importance of the genetic *v.* environmental contribution to both cannabis use and schizophrenia. This appears to vary across samples and is likely to depend on (a) the study sample size; a larger sample size is more likely to enable the detection of small effects and (b) the availability of cannabis and acceptability of its use during the period of data collection. For example, a recent study from the United States used data from twin pairs discordant for residential address with one twin living in a state where cannabis use was legal and the other twin living in a state where it was illegal. Their findings indicate that genetic correlations on frequency of cannabis use were significantly lower where cannabis use was legal compared to where it was illegal, suggesting the important influence of the social context (Zellers et al., 2023).

The EU-GEI sample is a unique sample with self-reported details on the type of cannabis used (Di Forti et al., 2019), which allowed us to explore for the first time the relationship between schizophrenia PRS and use of high-potency cannabis; the availability of which is increasing worldwide and is associated with high rates of psychosis across Europe (Di Forti et al., 2019). In our previous paper, we described a probabilistic sensitivity analysis, which showed that selection bias is unlikely to explain the reported findings on the observed magnitude of effect for daily cannabis use and use of high potency on the risk of psychotic disorder (Di Forti et al., 2019). The incorporation of frequency and potency of cannabis use alongside schizophrenia PRS results in an improvement in *R*^2^ values, indicating a significant improvement in the model's explanatory power. The delta *R*^2^, while positive, was modest, perhaps due to the multifactorial risk profile for psychotic disorders.

It should be noted that the estimates of cannabis potency cannot account for differences in the THC concentration in individual samples. Our dichotomous measure of THC above or below 10% is conservative and likely to have resulted in an underestimate of the effects of cannabis potency on the risk for psychotic disorder. We were not able to assess potency in UK Biobank. The people in UK Biobank were aged at least 40 years old at recruitment which was from 2006 to 2010, and most participants reported their last use of cannabis was well before recruitment (online Supplementary Fig. 6). There has been a significant change in the type of cannabis available in the UK in recent decades, and consequently, it is plausible that the middle-aged cannabis users within UK Biobank had mainly used lower potency cannabis (Potter et al., 2008, . 2018). While this lack of potency data is a limitation, it is worth noting that we still observe strong effects for frequency of use in the UK Biobank sample. If the cannabis used by many of these participants was indeed lower potency, it would follow that we might observe even stronger effects where the same analyses to be conducted with participants recruited today.

This study must be considered in the context of some limitations. First, the cannabis measures were based on retrospectively collected self-reported information. In both cohorts, the data on cannabis use were collected as part of a questionnaire which did not refer to the association between cannabis use and psychosis. Biological data on potency and accurate levels of THC can be obtained from blood samples and there is a validation of biological data only when measuring current use up to few weeks rather than lifetime use, which is the measure of exposure we use in our analyses. Furthermore, previous research has clearly reported the reliability of self-reported measures on the frequency and potency of the cannabis used (Bharat et al., 2023; Buchan, Dennis, Tims, & Diamond, 2002; Di Forti et al., 2009; Freeman et al., 2014).

In addition, individuals of European ancestry are over-represented in these analyses. We opted to utilize PRS-CSx, which allows the inclusion of multiple GWAS summary statistics and linkage disequilibrium (LD) reference panels to improve prediction in non-EUR populations. However, the predictive power of these PRS is limited by the available discovery datasets. This is a common problem in genetic studies, and one that must be rapidly addressed to ensure scientific discoveries, especially those with potential clinical implications, are relevant to all populations (Duncan et al., 2019; Fatumo et al., 2022; Peterson et al., 2019). The two samples included are very different, both in terms of recruitment strategy (case-control *v.* healthy volunteer) and the data collected, meaning that we could not carry out a direct replication across the two cohorts (Bycroft et al., 2018; Gayer-Anderson et al., 2020). A recent study investigated the impact of selection bias in the UK Biobank and found evidence that it can impact genetic correlation and Mendelian randomization results between several traits (Schoeler et al., 2023). This again indicates that replication of our findings is essential to draw firm conclusions applicable to a wider clinical and healthy control population.

Our analyses were adjusted for a range of demographic and genetic factors, aiming to account for potential confounding variables that could influence the association between schizophrenia PRS and cannabis use. One putatively confounding factor is the underlying genetic risk for CUD, which might drive cannabis use patterns and schizophrenia risk. We therefore additionally adjusted all models for a CUD PRS, to investigate evidence of confounding. In the EU-GEI sample, we show that CUD PRS is largely independently associated with schizophrenia risk and that the addition of this variable does not greatly impact the effect size for the schizophrenia PRS. This suggests that despite the known genetic overlap between the two traits, there is a degree of specificity in their association with schizophrenia risk. One speculative interpretation could be that a higher CUD PRS increases the likelihood of using cannabis, which in turn increases the risk for psychosis. If this were the case, we might expect to see evidence that adjusting for the CUD PRS reduces the effect of the measure of cannabis use frequency or potency, which was in fact not what we observed. Thus, we cannot rule out the possibility that some of the genetic factors that confer risk for CUD have a pleiotropic effect on schizophrenia pathogenesis.

Our work, along with multiple previous studies, confirms that cannabis use is much more common among people with psychosis than controls. We can, therefore, assume that a larger proportion of cases in the PGC and Genomic Psychiatry Cohort (GPC) GWAS on schizophrenia are cannabis users, relative to controls (Elkrief et al., 2023). This remains a limitation of genetic studies of cannabis and schizophrenia and it could potentially lead to an inflated estimate of the true shared genetic liability between CUD and schizophrenia. Future analyses would be improved by tracking down and accounting for comorbid cases when building schizophrenia GWASs (Colbert & Johnson, 2023). Finally, psychosis is a multifactorial disease with a wide number of established risk factors, not all of which can be accounted for in any single model. It remains possible that some of the findings detailed here could be explained, in part, by other factors such as comorbid disease, trauma, sociodemographic factors, or underlying genetic risk for other traits.

In conclusion, our findings indicate (a) heavy cannabis use remains a strong risk factor independent of schizophrenia genetic load and (b) as available samples increase for more well-powered and diverse GWASs on schizophrenia, schizophrenia PRS may be useful in identifying individuals most at risk for cannabis-associated psychosis (Pain & Lewis, 2022). Currently, schizophrenia PRS risk can only explain a small proportion of the risk (Power et al., 2014). While this study did not set out to prove causality or specifically address the above-mentioned methodological controversies around genetic confounding, it clearly shows that cannabis users at all levels of schizophrenia PRS are more likely to belong to the FEP group compared with non-cannabis-using individuals. This is consistent with the epidemiological evidence which shows that cannabis is an important and modifiable risk factor for psychosis, as it has recently been outlined by the World Federation Society of Biological Psychiatry (D'Souza et al., 2022). Therefore, our findings provide information that public education campaigns could use toward the prevention of an increase in the rates of psychotic disorders (Murray & Hall, 2020).

## Supporting information

Austin-Zimmerman et al. supplementary material Austin-Zimmerman et al. supplementary material

## Supplementary material

The supplementary material for this article can be found at https://doi.org/10.1017/S0033291724002058.

## References

[ref1] 1000 Genomes Project Consortium. (2010). A map of human genome variation from population scale sequencing. Nature, 467, 1061. doi:10.1038/nature09534 20981092 PMC3042601

[ref2] Agrawal, A., & Lynskey, M. T. (2006). The genetic epidemiology of cannabis use, abuse and dependence. Addiction, 101, 801–812. doi:10.1111/j.1360-0443.2006.01399.x 16696624

[ref3] Bharat, C., Webb, P., Wilkinson, Z., McKetin, R., Grebely, J., Farrell, M., … Clark, B. (2023). Agreement between self-reported illicit drug use and biological samples: A systematic review and meta-analysis. Addiction, 118, 1624–1648. doi:10.1111/add.16200 37005867

[ref4] Bigdeli, T. B., Genovese, G., Georgakopoulos, P., Meyers, J. L., Peterson, R. E., Iyegbe, C. O., … Pato, C. N. (2020). Contributions of common genetic variants to risk of schizophrenia among individuals of African and Latino ancestry. Molecular Psychiatry, 25, 2455–2467. doi:10.1038/s41380-019-0517-y 31591465 PMC7515843

[ref5] Brisacier, A.-C., Cadet-Taïrou, A., Díaz Gómez, C., Gandilhon, M., Le Nézet, O., & Lemenier-Jeannet, A. (2015). Drogues, chiffres clés. Paris: Observatoire Français des Drogues et des Toxicomanies. Government document. Retrieved from https://www.ofdt.fr/publications/collections/drogues-et-addictions-chiffres-cles/6eme-edition-2015/

[ref6] Buchan, B. J., Dennis, M. L., Tims, F. M., & Diamond, G. S. (2002). Cannabis use: Consistency and validity of self-report, on-site urine testing and laboratory testing. Addiction, 97(Suppl 1), 98–108. doi:10.1046/j.1360-0443.97.s01.1.x 12460132

[ref7] Bycroft, C., Freeman, C., Petkova, D., Band, G., Elliott, L. T., Sharp, K., … O'Connell, J. (2018). The UK Biobank resource with deep phenotyping and genomic data. Nature, 562, 203–209. doi:10.1038/s41586-018-0579-z 30305743 PMC6786975

[ref8] Callaghan, R. C., Sanches, M., Murray, R. M., Konefal, S., Maloney-Hall, B., & Kish, S. J. (2022). Associations between Canada's cannabis legalization and emergency department presentations for transient cannabis-induced psychosis and schizophrenia conditions: Ontario and Alberta, 2015–2019. Canadian Journal of Psychiatry, 67, 616–625. doi:10.1177/07067437211070650 35019734 PMC9301152

[ref9] Cheng, W., Parker, N., Karadag, N., Koch, E., Hindley, G., Icick, R., … Bahrami, S. (2023). The relationship between cannabis use, schizophrenia, and bipolar disorder: A genetically informed study. The Lancet. Psychiatry, 10, 441–451. doi:10.1016/S2215-0366(23)00143-8 37208114 PMC10311008

[ref10] Choi, S. (2020) GreedyRelated Project. Retrieved from https://gitlab.com/choishingwan/GreedyRelated

[ref11] Colbert, S. M. C., & Johnson, E. C. (2023). Genetic explanations for the association between cannabis and schizophrenia. In C. D. D'Souza, D. Castle, & Murray SR (eds.), Marijuana and madness (pp. 216–224). Cambridge, UK: Cambridge University Press.

[ref13] Demontis, D., Rajagopal, V. M., Thorgeirsson, T. E., Als, T. D., Grove, J., Leppälä, K., … Reginsson, G. W. (2019). Genome-wide association study implicates CHRNA2 in cannabis use disorder. Nature Neuroscience, 22, 1066–1074. doi:10.1038/s41593-019-0416-1 31209380 PMC7596896

[ref14] de Oliveira, G. L., Voloch, M. H., Sztulman, G. B., Neto, O. N., & Yonamine, M. (2008). Cannabinoid contents in cannabis products seized in São Paulo, Brazil, 2006–2007. Forensic Toxicology, 26, 31–35. doi:10.1007/s11419-008-0046-x

[ref16] Di Forti, M., Marconi, A., Carra, E., Fraietta, S., Trotta, A., Bonomo, M., … Russo, M. (2015). Proportion of patients in South London with first-episode psychosis attributable to use of high potency cannabis: A case-control study. The Lancet. Psychiatry, 2, 233–238. doi:10.1016/S2215-0366(14)00117-5 26359901

[ref15] Di Forti, M., Morgan, C., Dazzan, P., Pariante, C., Mondelli, V., Marques, T. R., … Paparelli, A. (2009). High-potency cannabis and the risk of psychosis. The British Journal of Psychiatry, 195, 488–491. doi:10.1192/bjp.bp.109.064220 19949195 PMC2801827

[ref17] Di Forti, M., Quattrone, D., Freeman, T. P., Tripoli, G., Gayer-Anderson, C., Quigley, H., … La Cascia, C. (2019). The contribution of cannabis use to variation in the incidence of psychotic disorder across Europe (EU-GEI): A multicentre case-control study. The Lancet. Psychiatry, 6, 427–436. doi:10.1016/S2215-0366(19)30048-3 30902669 PMC7646282

[ref18] D'Souza, D. C., DiForti, M., Ganesh, S., George, T. P., Hall, W., Hjorthøj, C., … Nguyen, T. B. (2022). Consensus paper of the WFSBP task force on cannabis, cannabinoids and psychosis. The World Journal of Biological Psychiatry, 23, 719–742. doi:10.1080/15622975.2022.2038797 35315315

[ref19] Duncan, L., Shen, H., Gelaye, B., Meijsen, J., Ressler, K., Feldman, M., … Domingue, B. (2019). Analysis of polygenic risk score usage and performance in diverse human populations. Nature Communications, 10, 3328. doi:10.1038/s41467-019-11112-0 PMC6658471 31346163

[ref12] Elkrief, L., Lin, B., Marchi, M., Afzali, M. H., Banaschewski, T., & Bokde, A. L. W., … consortium, I. (2023). Independent contribution of polygenic risk for schizophrenia and cannabis use in predicting psychotic-like experiences in young adulthood: Testing gene × environment moderation and mediation. Psychological Medicine, 53, 1759–1769. doi:10.1017/S0033291721003378 37310336 PMC10106286

[ref20] European Monitoring Centre for Drugs and Drug and Addiction (2016). European Drug report 2016: Trends and developments. Luxembourg: Publications Office of the European Union, 2016.

[ref21] Fatumo, S., Chikowore, T., Choudhury, A., Ayub, M., Martin, A. R., & Kuchenbaecker, K. (2022). A roadmap to increase diversity in genomic studies. Nature Medicine, 28, 243–250. doi:10.1038/s41591-021-01672-4 PMC7614889 35145307

[ref23] Freeman, T. P., Groshkova, T., Cunningham, A., Sedefov, R., Griffiths, P., & Lynskey, M. T. (2019). Increasing potency and price of cannabis in Europe, 2006–16. Addiction, 114, 1015–1023. doi:10.1111/add.14525 30597667 PMC6590252

[ref22] Freeman, T. P., Morgan, C. J., Hindocha, C., Schafer, G., Das, R. K., & Curran, H. V. (2014). Just say ‘know’: How do cannabinoid concentrations influence users' estimates of cannabis potency and the amount they roll in joints? Addiction, 109, 1686–1694. doi:10.1111/add.12634 24894801

[ref24] Gage, S. H., Hickman, M., & Zammit, S. (2016). Association between cannabis and psychosis: Epidemiologic evidence. Biological Psychiatry, 79, 549–556. doi:10.1016/j.biopsych.2015.08.001 26386480

[ref25] Gage, S. H., Jones, H. J., Burgess, S., Bowden, J., Davey Smith, G., Zammit, S., & Munafo, M. R. (2017). Assessing causality in associations between cannabis use and schizophrenia risk: A two-sample Mendelian randomization study. Psychological Medicine, 47, 971–980. doi:10.1017/S0033291716003172 27928975 PMC5341491

[ref26] Gayer-Anderson, C., Jongsma, H. E., Di Forti, M., Quattrone, D., Velthorst, E., De Haan, L., … Tortelli, A. (2020). The European Network of National Schizophrenia Networks Studying Gene–Environment Interactions (EU-GEI): Incidence and first-episode case–control programme. Social Psychiatry and Psychiatric Epidemiology, 55, 645–657. doi:10.1007/s00127-020-01831-x 31974809

[ref27] Ge, T., Chen, C.-Y., Ni, Y., Feng, Y.-C. A., & Smoller, J. W. (2019). Polygenic prediction via Bayesian regression and continuous shrinkage priors. Nature Communications, 10, 1776. doi:10.1038/s41467-019-09718-5 PMC6467998 30992449

[ref28] Gillespie, N. A., & Kendler, K. S. (2021). Use of genetically informed methods to clarify the nature of the association between cannabis use and risk for schizophrenia. JAMA Psychiatry, 78, 467–468. doi:10.1001/jamapsychiatry.2020.3564 33146687

[ref29] Gonçalves-Pinho, M., Bragança, M., & Freitas, A. (2020). Psychotic disorders hospitalizations associated with cannabis abuse or dependence: A nationwide big data analysis. International Journal of Methods in Psychiatric Research, 29, e1813. doi:10.1002/mpr.1813 31808250 PMC7051837

[ref30] Grucza, R. A., Agrawal, A., Krauss, M. J., Cavazos-Rehg, P. A., & Bierut, L. J. (2016). Recent trends in the prevalence of marijuana use and associated disorders in the United States. JAMA Psychiatry, 73, 300–301. doi:10.1001/jamapsychiatry.2015.3111 26864618 PMC5407184

[ref31] Guloksuz, S., Pries, L. K., Delespaul, P., Kenis, G., Luykx, J. J., Lin, B. D., … van Os, J. (2019). Examining the independent and joint effects of molecular genetic liability and environmental exposures in schizophrenia: Results from the EUGEI study. World Psychiatry, 18, 173–182. doi:10.1002/wps.20629 31059627 PMC6502485

[ref32] Henquet, C., Murray, R., Linszen, D., & van Os, J. (2005). The environment and schizophrenia: The role of cannabis use. Schizophrenia Bulletin, 31, 608–612. doi:10.1093/schbul/sbi027 15976013

[ref33] Hjorthøj, C., Posselt, C. M., & Nordentoft, M. (2021). Development over time of the population-attributable risk fraction for cannabis use disorder in schizophrenia in Denmark. JAMA Psychiatry, 78, 1013–1019. doi:10.1001/jamapsychiatry.2021.1471 34287621 PMC8295899

[ref34] Hjorthoj, C., Uddin, M. J., Wimberley, T., Dalsgaard, S., Hougaard, D. M., Borglum, A., … Nordentoft, M. (2021). No evidence of associations between genetic liability for schizophrenia and development of cannabis use disorder. Psychological Medicine, 51, 479–484. doi:10.1017/S0033291719003362 31813396

[ref35] Huang, J., Howie, B., McCarthy, S., Memari, Y., Walter, K., Min, J. L., … Zheng, H.-F. (2015). Improved imputation of low-frequency and rare variants using the UK10K haplotype reference panel. Nature Communications, 6, 8111. doi:10.1038/ncomms9111 PMC4579394 26368830

[ref36] Johnson, E. C., Demontis, D., Thorgeirsson, T. E., Walters, R. K., Polimanti, R., Hatoum, A. S., … Clarke, T.-K. (2020). A large-scale genome-wide association study meta-analysis of cannabis use disorder. The Lancet. Psychiatry, 7, 1032–1045. doi:10.1016/S2215-0366(20)30339-4 33096046 PMC7674631

[ref37] Jones, H. J., Hammerton, G., McCloud, T., Hines, L. A., Wright, C., Gage, S. H., … Zammit, S. (2022). Examining pathways between genetic liability for schizophrenia and patterns of tobacco and cannabis use in adolescence. Psychological Medicine, 52, 132–139. doi:10.1017/S0033291720001798 32515721 PMC7614952

[ref38] Jongsma, H. E., Gayer-Anderson, C., Lasalvia, A., Quattrone, D., Mulè, A., Szöke, A., … Tarricone, I. (2018). Treated incidence of psychotic disorders in the multinational EU-GEI study. JAMA Psychiatry, 75, 36–46. doi:10.1001/jamapsychiatry.2017.3554 29214289 PMC5833538

[ref39] Keller, M. C. (2014). Gene × environment interaction studies have not properly controlled for potential confounders: The problem and the (simple) solution. Biological Psychiatry, 75, 18–24. doi:10.1016/j.biopsych.2013.09.006 24135711 PMC3859520

[ref40] Kendler, K. S., Ohlsson, H., Maes, H. H., Sundquist, K., Lichtenstein, P., & Sundquist, J. (2015). A population-based Swedish Twin and Sibling Study of cannabis, stimulant and sedative abuse in men. Drug and Alcohol Dependence, 149, 49–54. doi:10.1016/j.drugalcdep.2015.01.016 25660314 PMC4431972

[ref41] Lee, S. H., Goddard, M. E., Wray, N. R., & Visscher, P. M. (2012). A better coefficient of determination for genetic profile analysis. Genetic Epidemiology, 36, 214–224. doi:10.1002/gepi.21614 22714935

[ref42] Lee Pow, J., Donald, C., di Forti, M., Roberts, T., Weiss, H. A., Ayinde, O., … Hutchinson, G. (2023). Cannabis use and psychotic disorders in diverse settings in the Global South: Findings from INTREPID II. Psychological Medicine, 53, 7062–7069. doi:10.1017/S0033291723000399 36951137 PMC10719629

[ref43] Levey, D. F., Galimberti, M., Deak, J. D., Wendt, F. R., Bhattacharya, A., Koller, D., … Gupta, P. (2023). Multi-ancestry genome-wide association study of cannabis use disorder yields insight into disease biology and public health implications. Nature Genetics, 55, 2094–2103. doi:10.1038/s41588-023-01563-z 37985822 PMC10703690

[ref44] Lewis, C. M., & Vassos, E. (2017). Prospects for using risk scores in polygenic medicine. Genome Medicine, 9, 96. doi:10.1186/s13073-017-0489-y 29132412 PMC5683372

[ref45] Mallett, R., Leff, J., Bhugra, D., Pang, D., & Zhao, J. H. (2002). Social environment, ethnicity and schizophrenia: A case-control study. Social Psychiatry and Psychiatric Epidemiology, 37, 329–335. doi:10.1007/s00127-002-0557-4 12111025

[ref46] Manichaikul, A., Mychaleckyj, J. C., Rich, S. S., Daly, K., Sale, M., & Chen, W.-M. (2010). Robust relationship inference in genome-wide association studies. Bioinformatics (Oxford, England), 26, 2867–2873. doi:10.1093/bioinformatics/btq559 20926424 PMC3025716

[ref47] Manthey, J. (2019). Cannabis use in Europe: Current trends and public health concerns. International Journal of Drug Policy, 68, 93–96. doi:10.1016/j.drugpo.2019.03.006 31030057

[ref48] Marconi, A., Di Forti, M., Lewis, C. M., Murray, R. M., & Vassos, E. (2016). Meta-analysis of the association between the level of cannabis use and risk of psychosis. Schizophrenia Bulletin, 42, 1262–1269. doi:10.1093/schbul/sbw003 26884547 PMC4988731

[ref49] McGuffin, P., Farmer, A., & Harvey, I. (1991). A polydiagnostic application of operational criteria in studies of psychotic illness: Development and reliability of the OPCRIT system. Archives of General Psychiatry, 48, 764–770. doi:10.1001/archpsyc.1991.01810320088015 1883262

[ref50] Moran, L. V., Tsang, E. S., Ongur, D., Hsu, J., & Choi, M. Y. (2022). Geographical variation in hospitalization for psychosis associated with cannabis use and cannabis legalization in the United States. Psychiatry Research, 308, 114387. doi:10.1016/j.psychres.2022.114387 35016118 PMC8833839

[ref52] Murray, R. M., Englund, A., Abi-Dargham, A., Lewis, D. A., Di Forti, M., Davies, C., … D'Souza, D. C. (2017). Cannabis-associated psychosis: Neural substrate and clinical impact. Neuropharmacology, 124, 89–104. doi:10.1016/j.neuropharm.2017.06.018 28634109

[ref51] Murray, R. M., & Hall, W. (2020). Will legalization and commercialization of cannabis use increase the incidence and prevalence of psychosis? JAMA Psychiatry, 77, 777–778. doi:10.1001/jamapsychiatry.2020.0339 32267480

[ref53] Niesink, R. J., Rigter, S., Koeter, M. W., & Brunt, T. M. (2015). Potency trends of Δ9-tetrahydrocannabinol, cannabidiol and cannabinol in cannabis in the Netherlands: 2005–15. Addiction, 110, 1941–1950. doi:10.1111/add.13082 26234170

[ref54] Pain, O. GenoPredPipe. https://github.com/opain/GenoPred/tree/master/GenoPredPipe.

[ref55] Pain, O., & Lewis, C. M. (2022). Using local genetic correlation improves polygenic score prediction across traits. bioRxiv. doi:10.1101/2022.03.10.483736

[ref56] Pain, O., Glanville, K. P., Hagenaars, S. P., Selzam, S., Fürtjes, A. E., Gaspar, H. A., … Lewis, C. M. (2021). Evaluation of polygenic prediction methodology within a reference-standardized framework. PLoS Genetics, 17, e1009021. doi:10.1371/journal.pgen.1009021 33945532 PMC8121285

[ref57] Pasman, J. A., Verweij, K. J., Gerring, Z., Stringer, S., Sanchez-Roige, S., Treur, J. L., … Ong, J.-S. (2018). GWAS of lifetime cannabis use reveals new risk loci, genetic overlap with psychiatric traits, and a causal effect of schizophrenia liability. Nature Neuroscience, 21, 1161–1170. doi:10.1038/s41593-018-0206-1 30150663 PMC6386176

[ref58] Peterson, R. E., Kuchenbaecker, K., Walters, R. K., Chen, C. Y., Popejoy, A. B., Periyasamy, S., … Duncan, L. E. (2019). Genome-wide association studies in ancestrally diverse populations: Opportunities, methods, pitfalls, and recommendations. Cell, 179, 589–603. doi:10.1016/j.cell.2019.08.051 31607513 PMC6939869

[ref59] Potter, D. J., Clark, P., & Brown, M. B. (2008). Potency of delta 9-THC and other cannabinoids in cannabis in England in 2005: Implications for psychoactivity and pharmacology. Journal of Forensic Sciences, 53, 90–94. doi:10.1111/j.1556-4029.2007.00603.x 18279244

[ref60] Potter, D. J., Hammond, K., Tuffnell, S., Walker, C., & Di Forti, M. (2018). Potency of delta(9)-tetrahydrocannabinol and other cannabinoids in cannabis in England in 2016: Implications for public health and pharmacology. Drug Testing and Analysis, 10, 628–635. doi:10.1002/dta.2368 29441730

[ref61] Power, R. A., Verweij, K. J., Zuhair, M., Montgomery, G. W., Henders, A. K., Heath, A. C., … Martin, N. G. (2014). Genetic predisposition to schizophrenia associated with increased use of cannabis. Molecular Psychiatry, 19, 1201–1204. doi:10.1038/mp.2014.51 24957864 PMC4382963

[ref62] Purcell, S., Neale, B., Todd-Brown, K., Thomas, L., Ferreira, M. A., Bender, D., … Daly, M. J. (2007). PLINK: A tool set for whole-genome association and population-based linkage analyses. American Journal of Human Genetics, 81, 559–575. doi:10.1086/519795 17701901 PMC1950838

[ref63] Quattrone, D., Di Forti, M., Gayer-Anderson, C., Ferraro, L., Jongsma, H. E., Tripoli, G., … Berardi, D. (2019). Transdiagnostic dimensions of psychopathology at first episode psychosis: Findings from the multinational EU-GEI study. Psychological Medicine, 49, 1378–1391. doi:10.1017/S0033291718002131 30282569 PMC6518388

[ref64] Quattrone, D., Reininghaus, U., Richards, A. L., Tripoli, G., Ferraro, L., Quattrone, A., … Gayer-Anderson, C. (2021). The continuity of effect of schizophrenia polygenic risk score and patterns of cannabis use on transdiagnostic symptom dimensions at first-episode psychosis: Findings from the EU-GEI study. Translational Psychiatry, 11, 423. doi:10.1038/s41398-021-01526-0 34376640 PMC8355107

[ref65] R Core Team. (2021). R: A language and environment for statistical computing. R Foundation for Statistical Computing.

[ref66] Robin, X., Turck, N., Hainard, A., Tiberti, N., Lisacek, F., Sanchez, J.-C., & Müller, M. (2011). pROC: An open-source package for R and S+ to analyze and compare ROC curves. BMC Bioinformatics, 12, 1–8. doi:10.1186/1471-2105-12-77 21414208 PMC3068975

[ref67] Rognli, E. B., Taipale, H., Hjorthøj, C., Mittendorfer-Rutz, E., Bramness, J. G., Heiberg, I. H., & Niemelä, S. (2023). Annual incidence of substance-induced psychoses in Scandinavia from 2000 to 2016. Psychological Medicine, 53, 5246–5255. doi:10.1017/S003329172200229X 35983644 PMC10476053

[ref68] Schoeler, T., Speed, D., Porcu, E., Pirastu, N., Pingault, J.-B., & Kutalik, Z. (2023). Participation bias in the UK Biobank distorts genetic associations and downstream analyses. Nature Human Behaviour, 7, 216–1227. doi:10.1038/s41562-023-01579-9 PMC10365993 37106081

[ref69] The Haplotype Reference Consortium. (2016). A reference panel of 64976 haplotypes for genotype imputation. Nature Genetics, 48, 1279–1283. doi:10.1038/ng.3643 27548312 PMC5388176

[ref70] Trubetskoy, V., Pardiñas, A. F., Qi, T., Panagiotaropoulou, G., Awasthi, S., Bigdeli, T. B., … Hall, L. S. (2022). Mapping genomic loci implicates genes and synaptic biology in schizophrenia. Nature, 604, 502–508. doi:10.1038/s41586-022-04434-5 35396580 PMC9392466

[ref71] UK10K Consortium. (2015). The UK10K project identifies rare variants in health and disease. Nature, 526, 82–90. doi:10.1038/nature14962 26367797 PMC4773891

[ref72] Vadhan, N. P., Corcoran, C. M., Bedi, G., Keilp, J. G., & Haney, M. (2017). Acute effects of smoked marijuana in marijuana smokers at clinical high-risk for psychosis: A preliminary study. Psychiatry Research, 257, 372–374. doi:10.1016/j.psychres.2017.07.070 28803095 PMC5890804

[ref73] Vaucher, J., Keating, B. J., Lasserre, A. M., Gan, W., Lyall, D. M., Ward, J., … Paré, G. (2018). Cannabis use and risk of schizophrenia: A Mendelian randomization study. Molecular Psychiatry, 23, 1287–1292. doi:10.1038/mp.2016.252 28115737 PMC5984096

[ref75] Verweij, K. J., Abdellaoui, A., Nivard, M. G., Cort, A. S., Ligthart, L., Draisma, H. H., … Hottenga, J.-J. (2017). Genetic association between schizophrenia and cannabis use. Drug and Alcohol Dependence, 171, 117–121. doi:10.1016/j.drugalcdep.2016.09.022 28086176 PMC5753881

[ref74] Verweij, K. J., Zietsch, B. P., Lynskey, M. T., Medland, S. E., Neale, M. C., Martin, N. G., … Vink, J. M. (2010). Genetic and environmental influences on cannabis use initiation and problematic use: A meta-analysis of twin studies. Addiction, 105, 417–430. doi:10.1111/j.1360-0443.2009.02831.x 20402985 PMC2858354

[ref76] Wainberg, M., Jacobs, G. R., di Forti, M., & Tripathy, S. J. (2021). Cannabis, schizophrenia genetic risk, and psychotic experiences: A cross-sectional study of 109308 participants from the UK Biobank. Translational Psychiatry, 11, 211. doi:10.1038/s41398-021-01330-w 33837184 PMC8035271

[ref77] Zamengo, L., Frison, G., Bettin, C., & Sciarrone, R. (2014). Cannabis potency in the Venice area (Italy): Update 2013. Drug Testing and Analysis, 7, 255–258. doi:10.1002/dta.1690 25069834

[ref78] Zellers, S. M., Ross, J. M., Saunders, G. R., Ellingson, J. M., Anderson, J. E., Corley, R. P., … McGue, M. K. (2023). Impacts of recreational cannabis legalization on cannabis use: A longitudinal discordant twin study. Addiction, 118, 110–118. doi:10.1111/add.16016 36002928 PMC10086942

