# Supplementary material for: The impact of schizophrenia genetic load and heavy cannabis use on the risk of psychotic disorder in the EU-GEI case-control and UK Biobank studies
Source: Psychol Med. 2024 Dec 6;54(15):4160–72. doi: 10.1017/S0033291724002058 (PMC11650186; doi:10.1017/S0033291724002058)
Supplement: Austin-Zimmerman et al. supplementary material [file S0033291724002058sup001.docx]

**Supplementary Material**

# Recruitment and cohort information: EU-GEI

First-episode psychosis patients (FEPp) were included if they were a) aged 18-64 years and b) resident within the study areas at the time of their first presentation, and received a diagnosis of psychosis (ICD-10 F20-29); further details are provided in previous publications (Di Forti et al., 2019; Quattrone et al., 2021). Using the Operational Criteria Checklist algorithm (McGuffin, Farmer & Harvey, 1991; Quattrone et al., 2019), all cases interviewed received a research-based diagnosis. FEPs were excluded if a) previously treated for psychosis, b) they met criteria for organic psychosis (ICD-10: F09), or for a diagnosis of transient psychotic symptoms resulting from acute intoxication (ICD-10: F1X.5). Random and Quota sampling strategies were adopted to guide the recruitment of controls from each of the sites. The most accurate local demographic data available were used to set quotas for controls to ensure the samples’ representativeness of each catchment area’s population at risk. Controls were excluded if they had received a diagnosis of, and/or treatment for, psychotic disorder.

*Cases*: We followed procedures previously used to generate representative samples of first-episode psychosis patients (FEPp) (Fearon et al., 2006). We identified all individuals aged 18 to 64 years, who contacted mental health services for a suspected first episode of psychosis (FEP), over periods up to four years in 17 catchment areas in England (Southeast London, Cambridgeshire & Peterborough); France (20^th^ arrondissement of Paris, Val-de-Marne, Puy-de-Dôme); the Netherlands (Central Amsterdam, Gouda & Voorhout); Italy (part of the Veneto region, Bologna, and Palermo); Spain (Madrid-Vallecas, Barcelona, Valencia, Oviedo, Santiago, Cuenca), and; Brazil (Ribeirão Preto, Sao Paulo) (full details of the incidence sample recruitment and general description of the incidence study methods are available in (Jongsma et al., 2018; Quattrone et al., 2019; Quattrone et al., 2021)).

Case ascertainment involved trained researchers making regular contact with all secondary and tertiary mental healthcare providers to identify potential cases and searching electronic clinical records, where available. In this process, all cases with psychosis within services were considered. In all countries, it was uncommon for people to be treated for FEP in primary care; instead, people with suspected psychosis would typically be referred to specialist mental health services. Research teams were overseen by a psychiatrist with experience in epidemiological research and included trained research nurses and clinical psychologists. Teams received training in epidemiological principles and incidence study design to minimize non-differential ascertainment bias across different local and national healthcare systems (see training package on the study website: ([https://www.kcl.ac.uk/ioppn/depts/hspr/research/social-epidemiology-research-group/current-projects.aspx](https://emea01.safelinks.protection.outlook.com/?url=https%3A%2F%2Fwww.kcl.ac.uk%2Fioppn%2Fdepts%2Fhspr%2Fresearch%2Fsocial-epidemiology-research-group%2Fcurrent-projects.aspx&data=01%7C01%7Cmarta.diforti%40kcl.ac.uk%7C122825d750d549175d9908d6488b8d57%7C8370cf1416f34c16b83c724071654356%7C0&sdata=ZFnLEzDAzK8NVlXuraP4pqgV%2FKpFPwT1DVIHePwld6E%3D&reserved=0)).

Between May 1 2010 and April 1 2015, we approached 1519 patients with first-episode psychosis. Of these 356 (1%) refused to participate, 19 (23%) could not consent because of language barriers and 14 (0.9%) were later excluded (London N=3; Madrid N=2; Bologna N=1; Ribeirão Preto N=8) as they did not meet the age inclusion criteria. For all patients who were not part of the study, local research ethics committees approved the extraction of demographics and clinical information from patient records. Patients who refused to participate were older [FEP_consented_ mean age = 30.8(10.5), median=29 (22 to 37); FEP_refused_ mean age= 32.8(11.5), median=31 (25 to 42); p<0.0001], more likely to be women [FEP_consented_ male = 558(61.9%); FEP_refused_ male 311 (54.7%), χ2(1)=7.6; p=0.00603] and of White European origin [χ2(5)=38, p<0.0001].

1130 First Episode Psychosis Patients (FEPp) across the study sites consented to take part in the case-control study (S-Table 1**)**. The FEPp recruited in the case-control study are broadly representative of the gender and ethnicity of the rest of the incidence sample. However, in London, Amsterdam and Ribeirao Preto cases aged 18-24 were over-represented in the case-control sample and those aged 45-54 and 55 or over were under-represented compared with the incidence sample (S-Table 2). (For a detailed recruitment flow chart for the EUGEI controls and cases see S-Figure 1 and S-Figure 2, respectively).

S-Table 1 Number of participants of the case-control study recruited by each site who met the inclusion criteria.

| Catchment area |  |  |
| --- | --- | --- |
| **England** | **Controls** | **Cases** |
| Southeast London | 230 | 201 |
| Cambridgeshire | 108 | 45 |
| **The Netherlands** |  |  |
| Amsterdam | 101 | 96 |
| Gouda & Voorhout | 109 | 100 |
| **Spain** |  |  |
| Madrid | 38 | 39 |
| Barcelona | 37 | 31 |
| Valencia* | 32 | 49 |
| Oviedo* | 39 | 39 |
| Santiago* | 38 | 28 |
| Cuenca* | 38 | 18 |
| **France** |  |  |
| Paris (Maison-Blanche)* | 0 | 36 |
| Paris (Val-de-Marne) | 100 | 54 |
| Puy-de-Dome | 47 | 15 |
| **Italy** |  |  |
| Bologna | 65 | 70 |
| Verona* | 115 | 59 |
| Palermo | 100 | 58 |
| **Brazil** |  |  |
| Ribeiãro Preto | 302 | 192 |
| **Total** | **1,499** | **1,130** |

*Sites excluded for the case-control analysis because of missing data =>10%. Mason-Blanche was excluded from the case-control analysis, as they did not recruit any controls.

*S-Table 2 χ^2^ and p-values for comparisons between those cases who participated in the case-control arm of the study and those who did not. The table shows how the case-control study cases are representative of the rest of the incidence sample by site. (Age range groups included the following categories: 18-24; 25-34; 35-44; 45-54; 55-64) (Jongsma, 2018)*

|  | **Age** | | | | **Sex** | | | |
| --- | --- | --- | --- | --- | --- | --- | --- | --- |
|  | Mean,sd; (Median) | | χ^2^ | p-value | Male %; N | | χ^2^ | p-value |
|  | case-control sample | rest of the incidence sample |  |  | case-control sample | rest of the incidence sample |  |  |
| **England** |  |  |  |  |  |  |  |  |
| Southeast London | 29·6,9·4 (27) | 34·6,11·2 (33) | **31·4** | **<0·01** | 63·2 (127) | 51·4 (112) | **5·9** | **0·02** |
| Cambridgeshire | 28·1,7·9 (26) | 32·5,12·3 (29) | 6·8 | 0·15 | 55·6 (25) | 57·0 (126) | 0·0 | 0·86 |
| **The Netherlands** |  |  |  |  |  |  |  |  |
| Amsterdam | 27·6,8·1 (25) | 38·2,12·5 (36) | **50·5** | **<0·01** | 74·0 (71) | 59·9 (118) | 5·6 | 0·18 |
| Gouda & Voorhout | 31.7,11·1 (29) | 32.5,12·0 (30) | 1 | 0·9 | 65·0 (65) | 54·6 (36) | 1·8 | 0·18 |
| **Spain** |  |  |  |  |  |  |  |  |
| Madrid | 33·1,11·1 (33) | 33·9,9·6 (30) | 2·5 | 0·64 | 69·2 (27) | 63·3 (31) | 0·3 | 0.56 |
| Barcelona | 29·4,11·3 (30) | 30·7,13·4 (28) | 2·5 | 0·63 | 74·2 (23) | 50·7 (39) | **5** | **0·02** |
| Valencia | 31·5,11·4 (27) | 35·6,10·3 (35·5) | 3·3 | 0·51 | 61·2 (30) | 20·0 (2) | **5·7** | **0·02** |
| Oviedo | 34·7,10·8 (35) | 36·0 9·7 (33) | 3·4 | 0·49 | 51·3 (20) | 46·5 (20) | 0·2 | 0·67 |
| Santiago | 32·1,11·2 (31) | 42·9,10·4 (44) | 8·7 | 0·07 | 64·3 (18) | 37·5 (3) | 1·8 | 0·17 |
| Cuenca | 29·2,9·5 (27) | 28·3,11·2 (25) | 0·7 | 0·88 | 77·8 (14) | 77·8 (7) | 0·0 | 1·00 |
| **France** |  |  |  |  |  |  |  |  |
| Paris (Maison-Blanche) | 31·4,10·2 (30) | 34·1,12·1 (31) | 2·9 | 0·56 | 66·7 (24) | 70·2 (59) | 0·1 | 0·69 |
| Paris (Val-de-Marne) | 31·3,10·1 (27) | 33.6, 11·2 (30) | 4·6 | 0·33 | 61·1 (33) | 48·1 (75) | 2·7 | 0·1 |
| Puy-de-Dome | 37·3,13·4 (32) | 33·7,12·7 (34) | 8·8 | 0·07 | 60·1 (9) | 70·4 (19) | 0·5 | 0·49 |
| **Italy** |  |  |  |  |  |  |  |  |
| Bologna | 32·5,9·9 (33) | 33·3,10·5 (30) | 7·2 | 0·13 | 50·0 (35) | 53·7 (51) | 0·2 | 0·64 |
| Veneto | 36·5,10·1 (37) | 36·6,12·3 (36·5) | 6·9 | 0·14 | 55·9 (33) | 52·0 (26) | 0·2 | 0·68 |
| Palermo | 30·1,8·9 (28) | 34·5,10·2 (31) | 12·7 | 0·01 | 58·6 (34) | 54·6 (66) | 0·3 | 0·6 |
| **Brazil** |  |  |  |  |  |  |  |  |
| Ribeirão Preto | 32·3,11·2 (30) | 35·9,10·6 (35) | **24·1** | **<0·01** | 56·8 (109) | 49·1 (161) | 2·9 | 0·09 |

*S-Figure 1 Recruitment flow chart for the EUGEI control population*


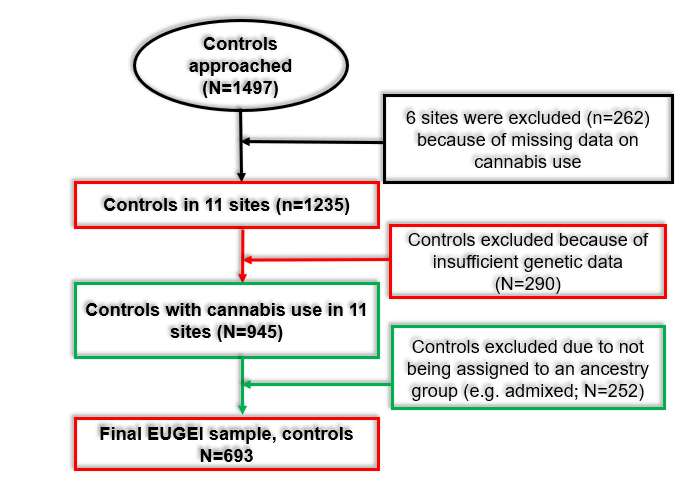


*S-Figure 2 Recruitment flow chart for the EUGEI FEPp cases*


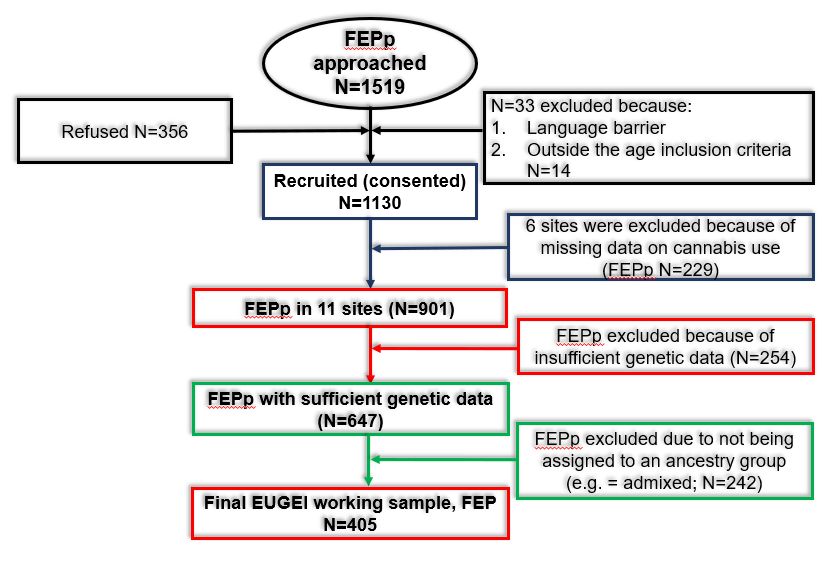


# Recruitment and cohort information: UK Biobank

Subjects were recruited from 22 UK assessment centres, with the requirement that participants should live within 10 miles of the centre. All subjects were aged between 40 and 70 years old and recruitment was conducted from 2006 to 2010. This study has been described in detail previously, see Bycroft *et al* (2018) (Bycroft et al., 2018). All necessary demographic, medical, and genetic data was downloaded from the UK Biobank.

Cases were defined as any participant with either a recorded diagnosis of psychotic disorder, identified through recorded ICD-10 data (codes F20-F29), or who self-reported “schizophrenia” or “Any other type of psychosis or psychotic illness” as part of the online MHQ (in response to the question: "Have you been diagnosed with one or more of the following mental health problems by a professional, even if you don't have it currently?"). Controls were defined as any UK Biobank participant who had no reported psychotic disorder or previous treatment with an antipsychotic. We compared the baseline demographic data as well as information on prescription of antipsychotics to consider the differences between cases defined by ICD-10 criteria and through self-report.

Data for a total of 455,538 UK Biobank participants with high-quality genetic data was downloaded. Of these, approximately 32% also responded to the MHQ and thus provided data on previous cannabis use, giving us a final working sample of 145,244 (N_EUR_= 143,600, N_AFR_=1,177, N_EAS_ = 527). Again, we were not able to draw meaningful conclusions from the non-European cohorts, given the relatively low numbers of cases among these populations.

Psychosis cases were defined either through ICD-10 clinical diagnosis (N=1,839) or by self-reported measures (N=524).

The final EUR sample consisted of 743 cases, as defined by a combination of ICD-10 data and self-report, and 142,857 controls (see S-Figure 3 for a detailed recruitment flow chart for the UK Biobank sample). As observed in the EU-GEI data, cases were on average younger and more likely to have used cannabis. Unlike EU-GEI, we did not observe a significant difference in sex between cases and controls (S-Table 3).

S-Table 3 Differences between cases* and controls across sociodemographic factors and patterns of cannabis use

|  |  | **UK Biobank** | | | | |
| --- | --- | --- | --- | --- | --- | --- |
|  |  | **Controls** | **Case^b^** | **df** | **Test stat** | **P** |
|  |  | (N=142,857) | (N=743) |  |  |  |
| **Age** | | 56.03 (7.71) | 54.66 (8.12) | 770.29 | t = 4.66 | 3.76E-06 |
| mean (SD) | |  |  |  |  |  |
| **Gender** | | 62,235 (43.56) | 325 (43.74) | 1 | X^2^ = 0.01 | 0.92 |
| N male (% male) | |  |  |  |  |  |
| **Lifetime cannabis use** | | 30,855 (21.60) | 254 (34.19) | 1 | X^2^ = 68.46 | <2.2x10^-16^ |
| N used (% used) | |  |  |  |  |  |
| **Frequency of use^e^ N (%)** | Never used | 112,002 (78.40) | 489 (65.81) | 5 | X^2^ = 72.07 | 3.8x10^-14^ |
|  | Rare use | 19,601 (13.72) | 126 (16.96) |  |  |  |
|  | < 1/ week | 3,557 (2.49) | 21 (2.83) |  |  |  |
|  | Weekly | 4,839 (3.39) | 55 (7.40) |  |  |  |
|  | Daily | 1,928 (1.35) | 46 (6.19) |  |  |  |

*Df = Degrees of freedom; SD= Standard deviation; THC = Tetrahydrocannabinol; ^a^ Cases for EU-GEI study defined as first episode psychosis patients; Cases for UK Biobank defined as either ^b^ Schizophrenia or psychosis based on self-report and/or ICD-10 code or ^c^ Any major psychiatric disorder defined by ICD-10 code; ^d^ EU-GEI study recorded data on age at first use, UK Biobank recorded data on age at last use;*

S-Figure 3 Recruitment flow chart for the UK Biobank cohort


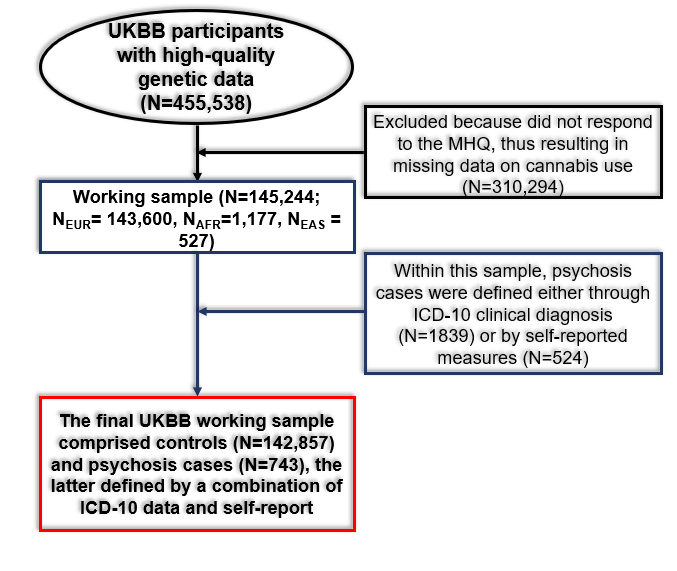


## UK Biobank ICD-10 psychosis cases versus self-report psychosis cases

Among the self-report cases the schizophrenia PRS explains a very small and non-significant proportion of the variance in case/control status (see below). This has been previously reported and thus we wanted to consider the potential sociodemographic differences between cases defined through ICD-10 criteria and those defined as self-report cases to determine why this might be. Those have responded to the MHQ and are considered ‘self-report’ cases only are younger, more likely to be female, and less likely to be taking an antipsychotic at time of recruitment (S-Table 4). There were similarities in the range of medications reported by ICD-10 cases and self-report cases, with olanzapine being the most frequently reported medication in both groups (S-Figure 4).

S-Table 4 Demographic comparison of cases defined through ICD-10 diagnosis and those defined by self-report measures.

|  | **ICD-10** | **Self-report** | **Df** | **Test stat** | **P** |
| --- | --- | --- | --- | --- | --- |
|  | **(N=1839)** | **(N=524)** |  |  |  |
| Age: | 55.9 (8.4) | 54.9 (8.0) | 880.4 | T = 2.4 | 0.02 |
| Mean (SD) |  |  |  |  |  |
| Sex: | 955 (51.9) | 216 (41.1) | 1 | Χ^2^ = 18.3 | 1.9x10^-5^ |
| N Male (%) |  |  |  |  |  |
| Ethnicity: | EUR: 1,717 (93.4) | EUR: 512 (97.7) | 2 | Χ^2^ = 14.4 | 0.001 |
| N (%) | EAS: 11 (0.6) | EAS: 1 (0.2) |  |  |  |
|  | AFR: 111 (6.0) | AFR: 11 (2.1) |  |  |  |
| Takes antipsychotic: | 761 (41.4) | 110 (21.0) | 1 | Χ^2^ = 72.0 | <2.2x10^-16^ |
| N (%) |  |  |  |  |  |
| **Lifetime cannabis use^a^** | 73 (30) | 187 (55) | 1 | X^2^ =2 | 0.1 |
| N used (% used) |  |  |  |  |  |

S-Figure 4 Comparison of the frequency of reported medications between psychosis cases defined through ICD-10 diagnosis and those defined by self-report measures.

*
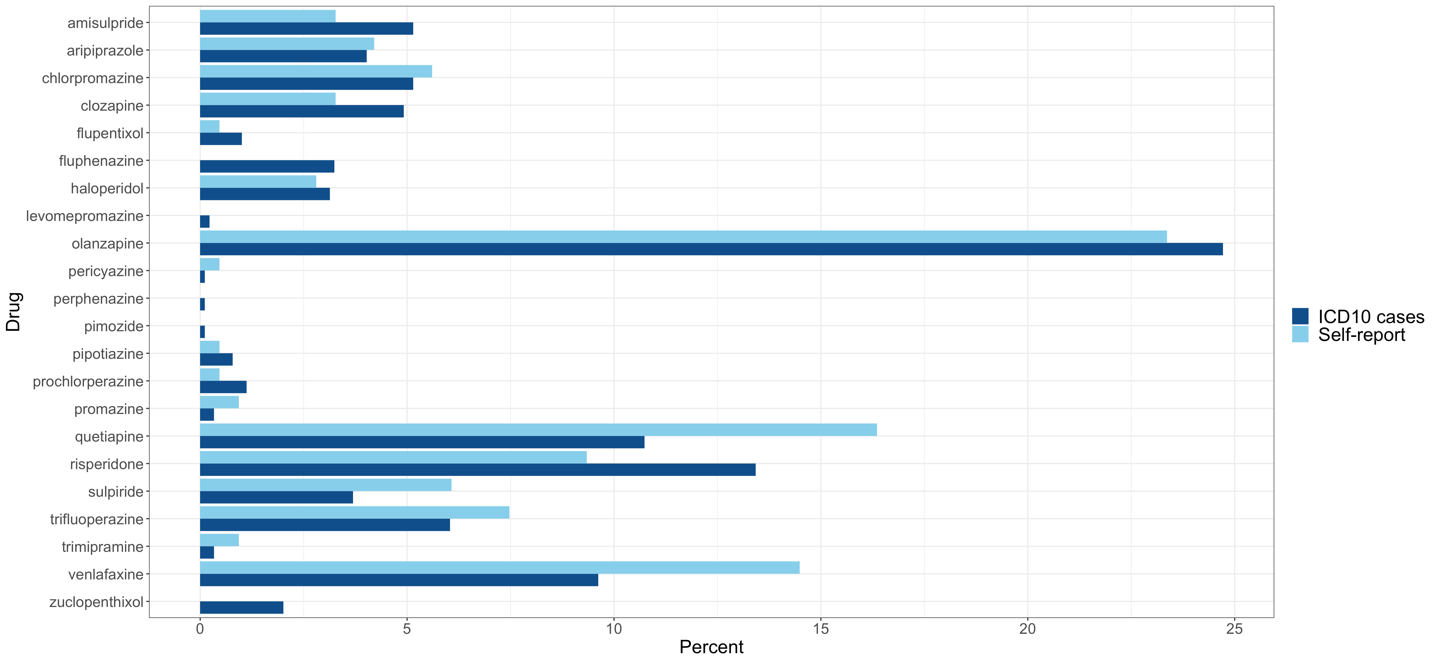
*

# *Measures of cannabis use: EU-GEI*

Data on patterns of cannabis use were collected in the EU-GEI study using the modified Cannabis Experience Questionnaire further updated (CEQ_EU-GEI_) (Di Forti et al., 2019). None of the materials used for the participants' recruitment referred to cannabis or to its potential role as a risk factor for psychotic disorder. Participants were asked if they had ever used cannabis. If yes, they were asked to answer questions about their pattern of use, including the type of cannabis allowing the participants to report the “street” name, in the original language, of the cannabis they used with no reference to its potency.

We used measures of cannabis use that we previously reported to increase the odds ratio for psychotic disorder (in an independent sample) (Di Forti et al., 2015): 1) Age at first use of cannabis; 2) lifetime frequency of use and 3) the potency of the cannabis used. The latter was estimated, as described in Di Forti *et al (Di Forti et al., 2019)* using the EMCDDA 2016 report (European Monitoring Centre for Drugs and Drug and Addiction (EMCDDA), 2016) and additional National published data on the concentration (%) of Tetrahydrocannabinol (THC) expected in the different types of cannabis available across Europe (Brisacier et al., 2015; de Oliveira, Voloch, Sztulman, Neto & Yonamine, 2008; European Monitoring Centre for Drugs and Drug and Addiction (EMCDDA), 2016; Hardwick & King, 2008; Niesink, Rigter, Koeter & Brunt, 2015; Potter, Clark & Brown, 2008; Potter, Hammond, Tuffnell, Walker & Di Forti, 2018; Zamengo, Frison, Bettin & Sciarrone, 2014) (see supplementary material).

We also kept the variable ‘lifetime’ ever cannabis use Yes/No to be able to compare our findings to the existing literature on the genetics of cannabis initiation (Pasman et al., 2018). Finally, we used the lifetime frequency of use and the cannabis potency variables to build the “*frequency-type* composite cannabis use measure” that we previously found (Di Forti et al., 2015) (and replicated (Di Forti et al., 2019)) to be a strong predictor of psychotic disorder. Low-potency cannabis was defined as cannabis with a THC concentration of less than 10%, and high-potency cannabis was defined as THC concentration greater or equal to 10%. The ‘*frequency-potency’* composite cannabis use measure includes seven scores associated with a steady (from 0 to 6) increase in the odds ratio for psychotic disorder (Di Forti et al., 2015; Di Forti et al., 2019): never used cannabis= 0; rare use of low potency cannabis =1; rare use of high potency cannabis =2; weekly use low potency cannabis =3; weekly use of high potency cannabis =4; daily use of low potency cannabis =5; daily use of high potency cannabis =6.

The cannabis potency variable was standardised based on the official home office data from the different countries, which is the most reliable measure of the type of street cannabis available at the time of the study. It was created using a cut-off of THC=10% based on the mean THC concentration expected in the different types of cannabis available across the side sites, as reported in the EMCDDA and by the National data on cannabis potency quoted (European Monitoring Centre for Drugs and Drug and Addiction (EMCDDA), 2016). Participants were asked to name in their own language the name of the type of cannabis they mostly used during their period of use.

The low-potency cannabis category (THC<10%) included hash/resin from the UK and Italy, imported herbal cannabis from UK, Italy, Spain and France, Brazilian marijuana and hash and the Dutch Geimporteerde Wiet. The high-potency category (THC=>10%) included all the other types reported by the study participants in their original language street names such as: UK home-grown skunk/sensimilla UK Super Skunk, Italian home-grown skunk/sensimilla, Italian Super Skunk, the Dutch Nederwiet, Nederhasj and geimporteerde hasj, the Spanish and French Hashish (from Morocco), Spanish home-grown sensimilla, French home-grown skunk/sensimilla/super-skunk and Brazilian skunk (Brisacier et al., 2015; de Oliveira et al., 2008; Hardwick & King, 2008; Niesink et al., 2015; Potter et al., 2008; Potter et al., 2018; Zamengo et al., 2014).

# *Measures of cannabis use: UK Biobank*

In the UK Biobank sample, three specific questions on cannabis use were recorded as part of the online mental health questionnaire. The first question was “*Have you taken CANNABIS (marijuana, grass, hash, ganja, blow, draw, skunk, weed, spliff, dope), even if it was a long time ago?*”, which was posed to 157,317 subjects. Those who answered yes were subsequently asked “C*onsidering when you were taking cannabis most regularly, how often did you take it?*” and “*About how old were you when you last had cannabis?*”. We used this data to identify those subjects that had a) never used cannabis, b) used cannabis at least once, c) used cannabis weekly at some stage, d) used cannabis daily at some stage. Data on potency or age of first use was not captured.

Given the cross-sectional nature of the UK Biobank study, it is not always possible to establish temporality in recorded variables. In the case of cannabis use, however, participants were specifically asked the age when they last used the substance. This confirms that the majority of participants last used cannabis well before they were recruited to the study (given the lower age limit for recruitment is 40 years old), with the mean age at last use 33.93±13.62 among cases and 32.28±12.91 among controls (t=-1.93, df = 256.8, p=0.06) (S-Figure 5).

S-Figure 5 Reported age of last cannabis use among cases and controls in the UK Biobank sample

*
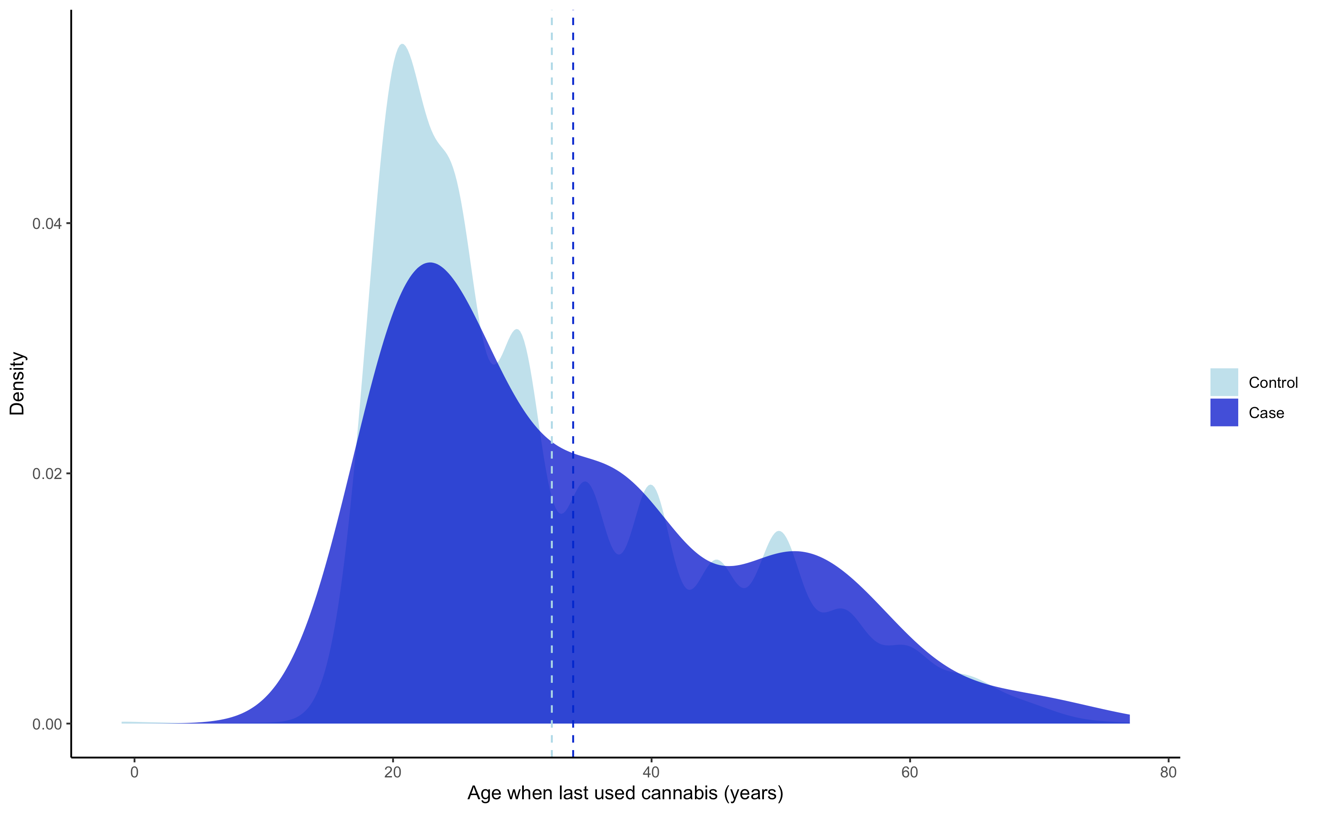
*

# PRS Optimisation

Polygenic risk scores (PRS) for schizophrenia in European ancestry participants were generated using PRS-CS (Ge, Chen, Ni, Feng & Smoller, 2019) and Plink (Purcell et al., 2007) based on GWAS summary statistics from the Schizophrenia Working Group of the Psychiatric Genomics Consortium (PGC) wave three (Ripke et al., 2014; Schizophrenia Working Group of the Psychiatric Genomics Consortium, 2020). These summary statistics include individuals of both European (EUR) East Asian (EAS) ancestry. We compared the PRS calculated using these summary statistics to a version using EUR only and demonstrate that the inclusion of EAS improves model prediction (S-Figure 6).

S-Figure 6 Predictive power of different polygenic risk score methods in the EU-GEI sample.


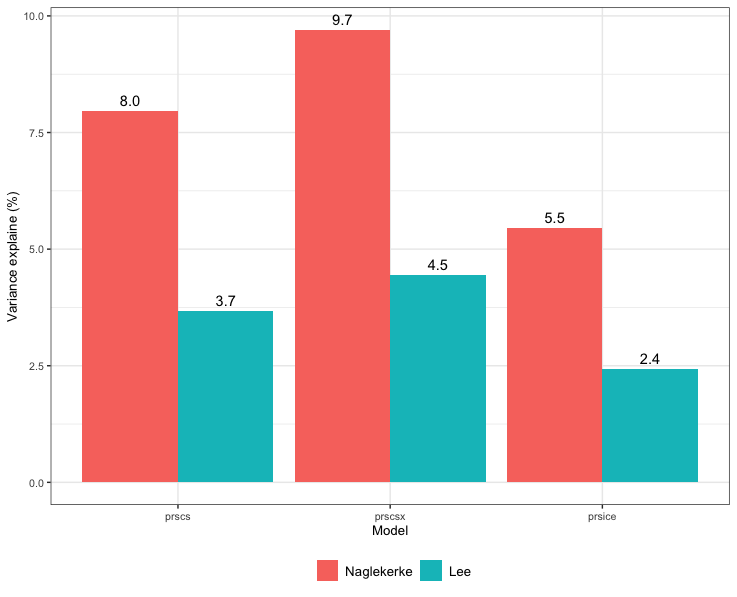


For individuals of African ancestry, we used PRS-CSx (Ge et al., 2022; Ruan et al., 2022), an extension of PRS-CS that jointly models GWAS summary statistics and LD reference data from different populations to improve polygenic prediction. The use of a shared continuous shrinkage prior improves effect size estimates by accounting for differences in allele frequency and patterns of linkage disequilibrium. AFR PRS were based on summary statistics from the Genomic Psychiatry Cohort, which includes 6,152 cases and 3,918 screen controls of admixed African ancestry. Each PRS was standardised to mean of 0 and standard deviation of 1 (Lewis & Vassos, 2017). We compared these PRS to PRS previously calculated using PRSice and demonstrated improved model prediction using PRS-CS and PRS-CSx compared to PRSice.

# *Polygenic risk score distribution: Schizophrenia PRS*

As reported in the main manuscript, the schizophrenia PRS was on average higher in first episode psychosis patients than in controls in the EU-GEI cohort (Figure 1).

The mean PRS was higher among cases compared to controls. UK Biobank case mean schizophrenia PRS=0.57/0.17, SD=0.24/0.93; controls mean schizophrenia PRS=0.50/-0.09, SD=0.24/0.93; t=-7.75, df=763.69; p=2.99x10^-14^. We see more controls in the schizophrenia PRS quintile 1 compared to cases, while the opposite was true in quintile 5: UKBB controls quintile 1=28,214/28,312 (%); UKBB cases quintile 1=98/28,312(%); controls quintile 5=28,094/28,312 (%); cases quintile 5=218/28,312 (%) (X^2^=53.19, df=4, p=7.79x10^-11^) (S-Figure 7).

S-Figure 7 Distribution of schizophrenia PRS in UK Biobank participants with and without a history or psychosis


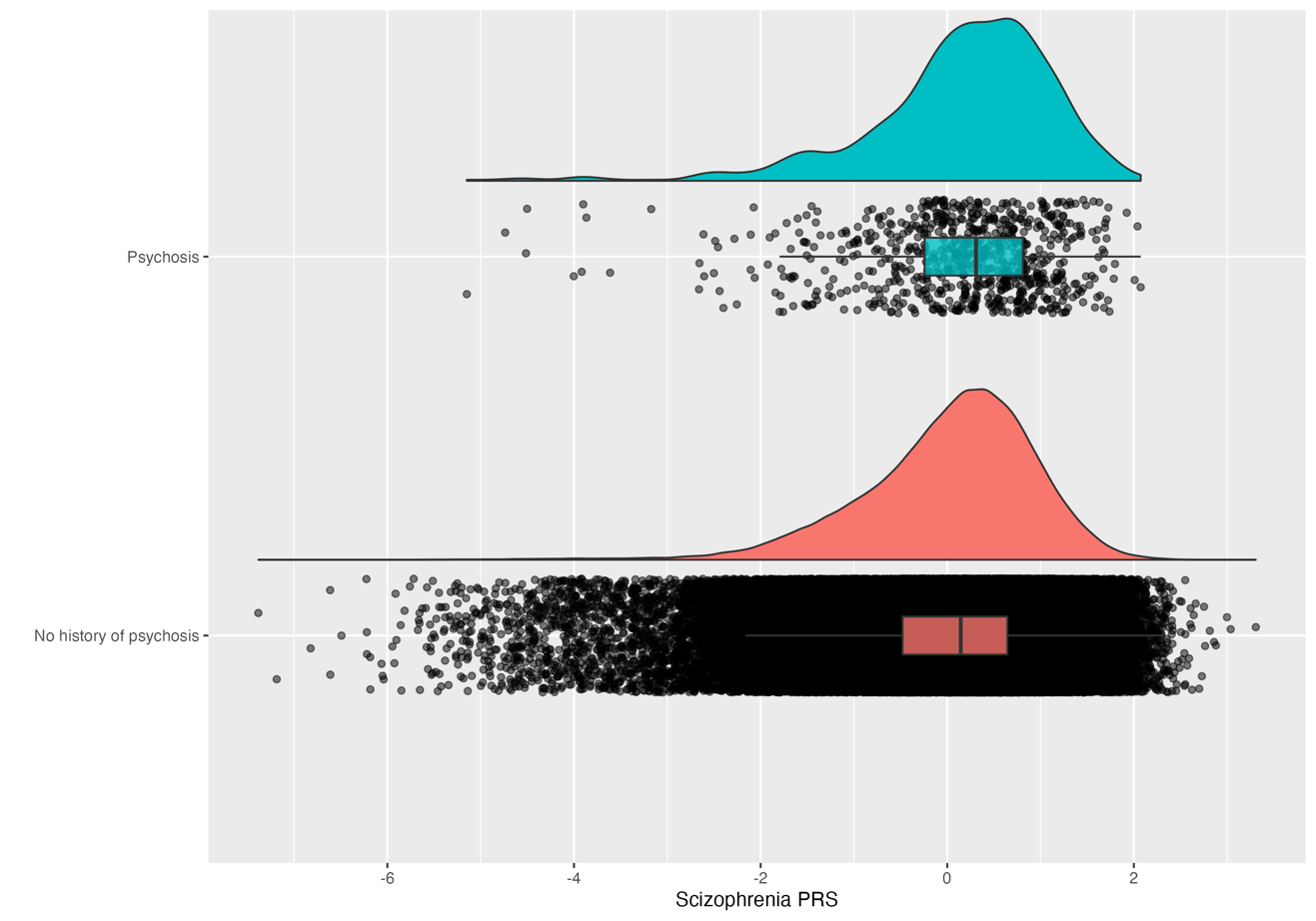


# *Polygenic risk score distribution: Cannabis use disorder PRS*

The CUD PRS was also higher in EU-GEI first episode psychosis cases compared to controls: EU-GEI case mean PRS = 0.31±1.1, controls mean CUD PRS = -0.1±0.98, p diff = 7.63e-14) (S-Figure 8). The CUD PRS was on average higher in participants with psychosis compared to those without (mean PRS psychosis = 0.04±1, mean PRS without psychosis = 0.04±0.93, p diff = 8e-5) (S-Figure 9).

S-Figure 8 Distribution of cannabis use disorder PRS among EU-GEI FEP cases (blue) and controls (red)


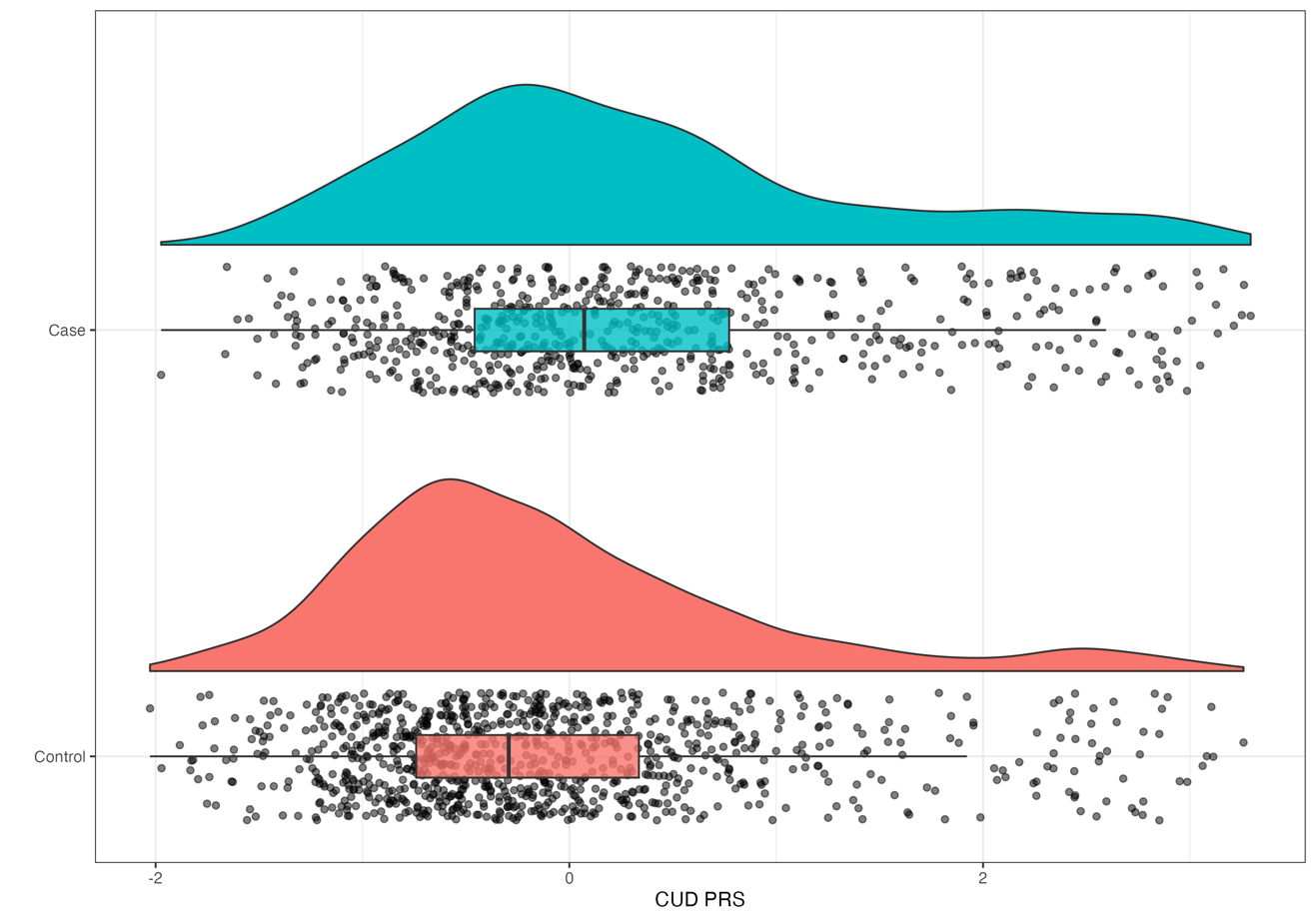


S-Figure 9 Distribution of cannabis use disorder PRS among UK Biobank participants with psychosis (blue) and UK Biobank participants with no history of psychosis (red)

#
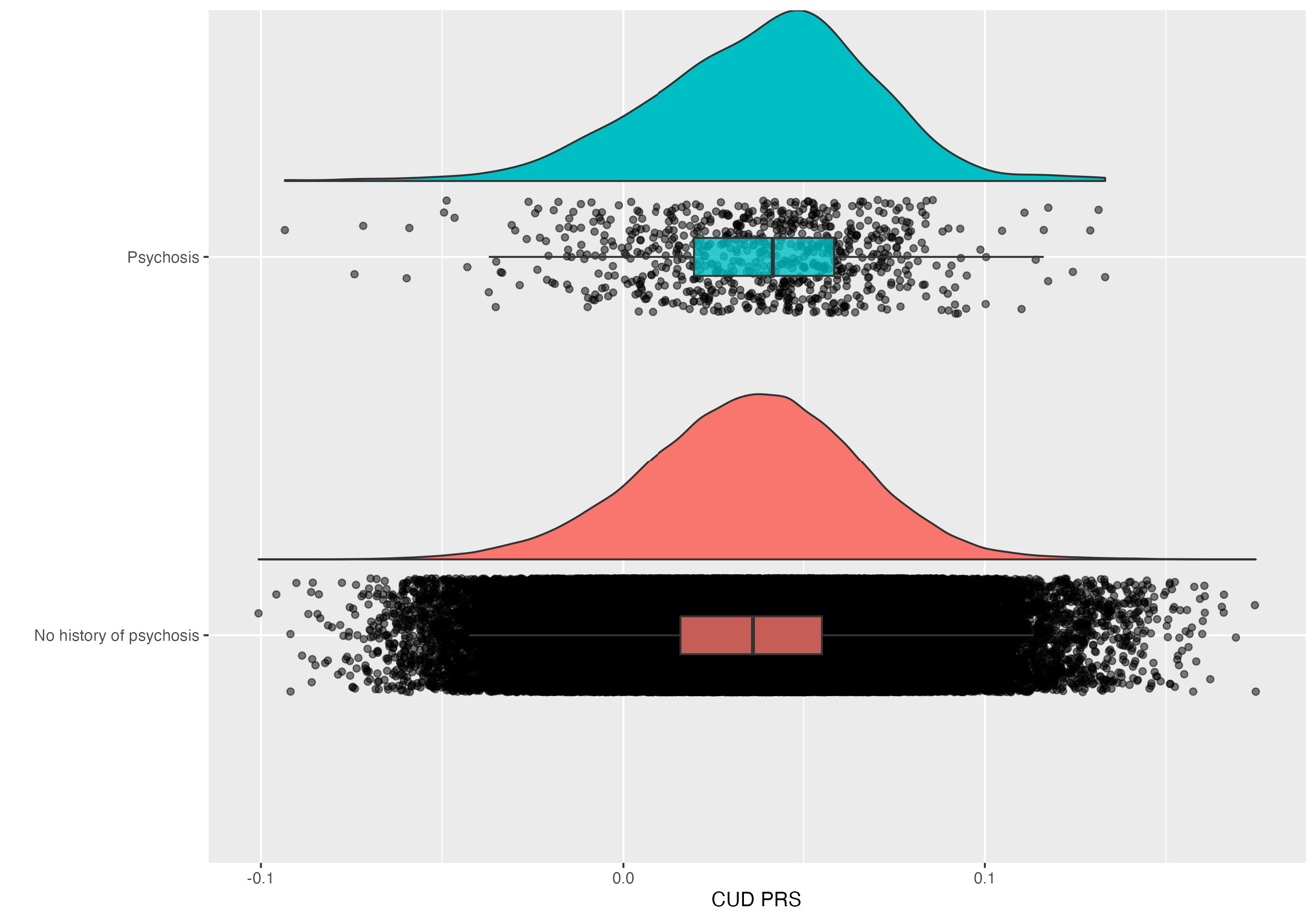
*Proportion of the variance explained between cases and controls by patterns of cannabis use and schizophrenia PRS*

## *EU-GEI*

We calculated pseudo-R^2^ statistics (Nagelkerke and liability scale, adjusted for the sample prevalence) by schizophrenia PRS in each ancestry group within EU-GEI: EUR (Nagelkerke R^2^=13.4%, Liability scale R^2^=5.98%; p=6.2x10^-13^), AFR (Nagelkerke R^2^=4.1%, Liability scale R^2^=1.8%; p=0.445), and EAS (Nagelkerke R^2^=19.6%, Liability scale R^2^=10.5%; p=0.311). These differences in R^2^ across the three main ethnic groups reflect the over-representation of individuals of European Ancestry in the PGC3 training sample used to calculate the schizophrenia PRS and are consistent with previous reports (Vassos et al., 2017).

In the EU-GEI cohort, a model including only site, sex, and 10 principal components explained 11.7% of the variance between cases and controls (5.3% on the liability scale). Adding age increased this to 17.0% (8.0% on the liability scale).

Adding the schizophrenia PRS increased this still further to 25.9% (12.8% on the liability scale). When tobacco smoking (more or less than 10 cigarettes per day) was included, the variance explained was 34.2% on the observed and 18.0% on the liability scale. Adding daily cannabis use increased this to 50.3% on the observed and 29.8% on the liability scale. This was not significantly increased by adding age at first use (R2obs = 50.3%, R2liab = 30.1%) but was increased by adding the use of high potency cannabis (THC > 10%) (R2obs = 55.5%, R2liab=34.3%). Adding the CUD PRS did not improve the model (R2obs = 56.0%, R2liab = 34.7%). (S-figure 10).

## *UK Biobank*

We calculated the R^2^ (both Nagelkerke and liability scale (Lee, Goddard, Wray & Visscher, 2012), adjusted for sample prevalence) pf schizophrenia case/control status as predicted by the schizophrenia PRS in each of our main ancestry groups within UK Biobank: European (Nagelkerke R^2^=3.56%, liability scale R^2^=6.01%, p=3.4x10^-12^), African (Nagelkerke R^2^=1.55%, Lee R^2^=2.62%, p=0.011), and East Asian (Nagelkerke R^2^=6.4%, liability scale R^2^=11.0%, p=2.6x10^-30^).

In the UKB cohort, a model including only site, sex, and 10 principal components plus age, sex and sites explained 0.7% of the variance between cases and controls (1.16% on liability scale). Adding age increased this to 0.92% (1.54% on the liability scale).

Adding the schizophrenia PRS increased this still further to 1.31% (2.2% on the liability scale). When tobacco smoking (more or less than 10 cigarettes per day) was included, the variance explained was 1.72% on the observed and 2.87% on the liability scale. Adding daily cannabis use increased this to 29.29% on the observed and 48.28% on the liability scale. CUD PRS was not associated with schizophrenia status and did not increase the variance explained 29.3% (48.29% on the liability scale) (S-figure 11).

In our UK Biobank control sample alone, schizophrenia PRS and 10 principal components explained 0.8% of the variance between those who never used cannabis and tried it at least once (p SCZ PRS<2.2x10^-16^) and 0.8% of the variance between those who never used and used daily having used it daily (p SCZ PRS=7.83x10^-6^).

S-Figure 10 Variance explained by nested models predicting psychosis case-control status in the EU-GEI cohort. Models numbered 1:7 including the following variables: 1) sex, site, 10 principal components, 2) adding age, 3) adding schizophrenia PRS, 4) adding tobacco smoking, 5) adding daily cannabis use, 6) adding age of first cannabis use, 7) adding potency of THC.


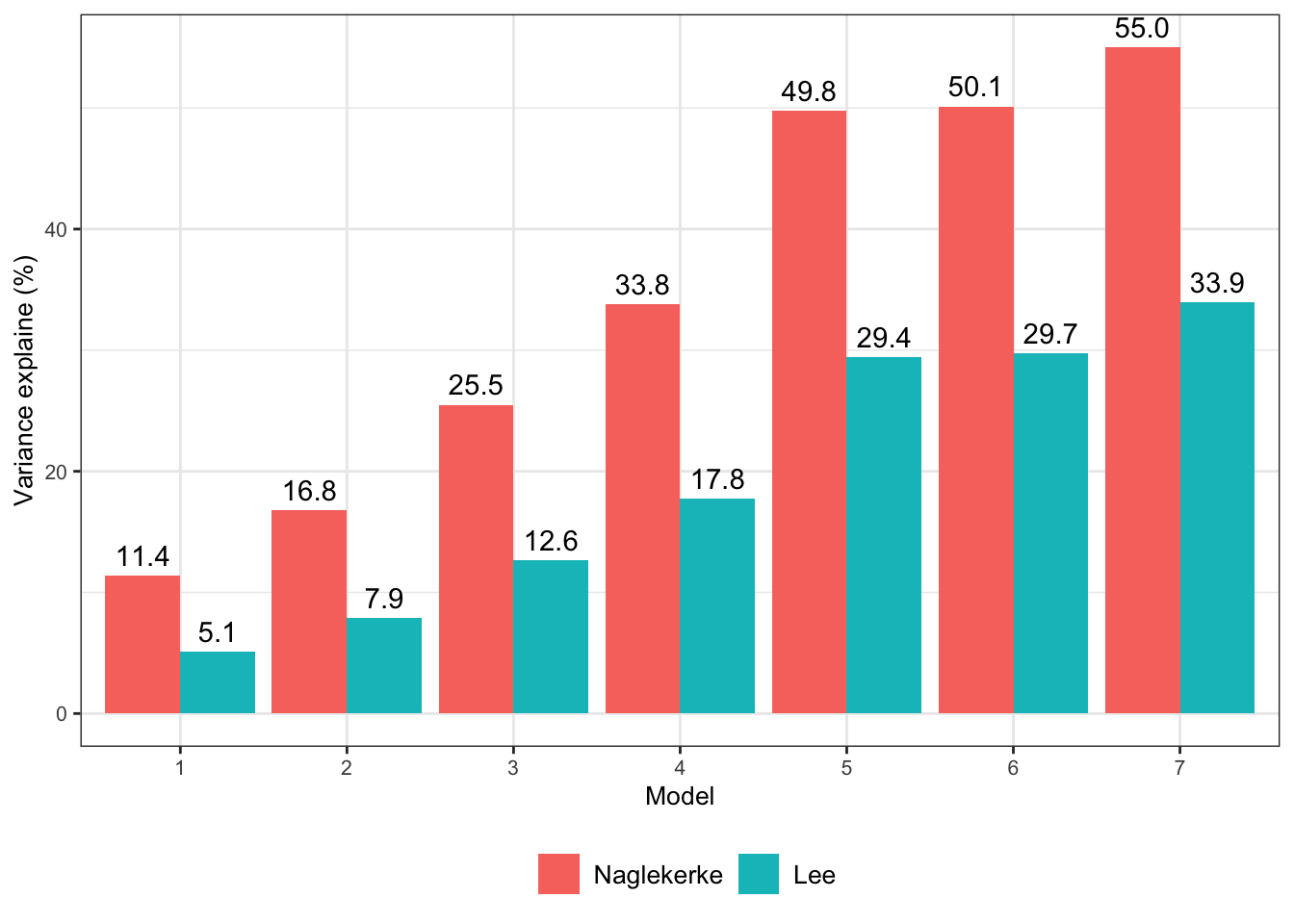


S-Figure 11 Variance explained by nested models predicting psychosis status in the UK Biobank cohort. Models numbered 1:6 including the following variables: 1) sex, site, 10 principal components, 2) adding age, 3) adding schizophrenia PRS, 4) adding tobacco smoking, 5) adding daily cannabis use, 6) adding age of first cannabis use, 7) adding CUD PRS.


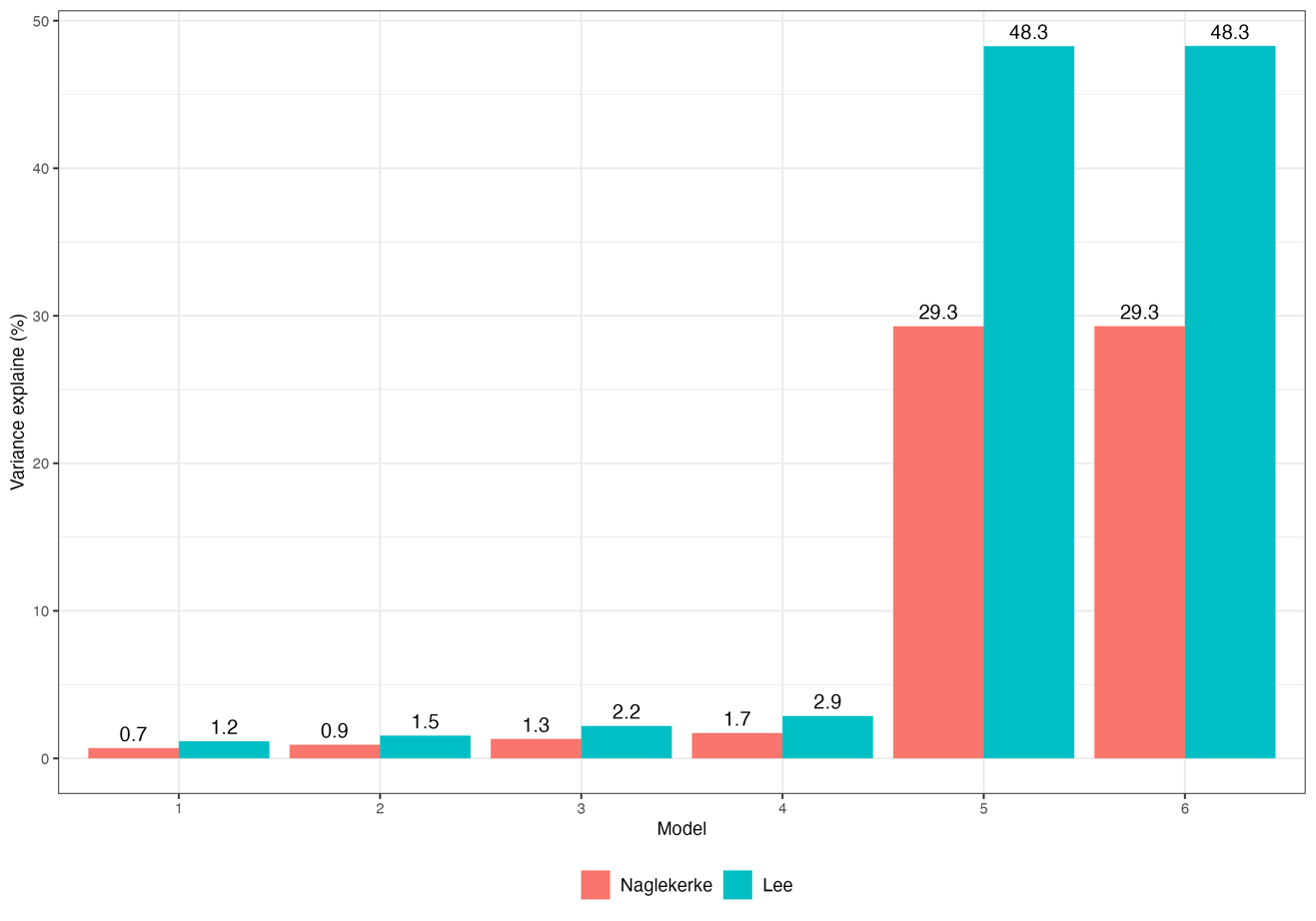


# *THC potency*

Using data from the EU-GEI sample only, we investigated the impact of high-potency THC on psychosis risk. In this sample, we found that those who reported using more potent types of cannabis (THC ≥ 10%) were more likely to develop psychosis than those who reported using lower potency cannabis. This was true both when adjusting for schizophrenia PRS and not (adjusted OR =1.84; 95% CI = 1.23:2.78; p=3.38x10^-3^; crude OR = 1.83; 95% CI = 1.24:2.72; p=0.003). In addition, users of both low and high-potency THC cannabis were more likely to develop psychosis than non-users, with a greater effect for high-potency cannabis users (low potency: adjusted OR =1.34; 95% CI = 0.99:1.81; p=0.06; crude OR = 1.38; 95% CI = 1.03:1.85; p=0.03; high potency: adjusted OR =2.56; 95% CI = 1.81:3.63; p=1.3x10^-7^; crude OR = 2.57; 95% CI = 1.84:3.61; p=3.81x10^-8^) (S-Tables 5, 6, and 7)

S-Table 5 The effect of THC potency on risk of psychosis in the EU-GEI sample. All models adjusted for age, sex, recruitment site, tobacco smoking, 10 principal components.

|  |  | | | **High or low potency (users only)** | | |  | **Use of high or low potency compared to never use** | | | |
| --- | --- | --- | --- | --- | --- | --- | --- | --- | --- | --- | --- |
| *Predictors* | | *N* | *Odds Ratios* | | *CI* | *P* | | *N* | *Odds Ratios* | *CI* | *p* |
| Low potency^*^ cannabis use | | *763* | *Reference* | | | | | 1,472 | 1.34 | 0.99: 1.81 | 0.060 |
| High potency^*^ cannabis use | |  | 1.84 | | 1.23: 2.78 | **3.38x10^-3^** | |  | 2.56 | 1.81: 3.63 | **1.30x10^-7^** |
| Schizophrenia PRS | |  | 2.92 | | 2.15: 4.03 | **2.40x10^-11^** | |  | 2.91 | 2.34: 3.64 | **2.34x10^-21^** |

^*^ *Low potency cannabis = THC concentration <10%; High potency cannabis = THC concentration ≥10%.*

S-Table 6 Assessing the interaction between frequency and potency of cannabis use in the EU-GEI EUR-only sample. All models adjusted for age, sex, recruitment site, tobacco smoking, 10 principal components.

|  | Frequency/potency composite measure (N=728) | | | |
| --- | --- | --- | --- | --- |
| *Predictors* | *Odds Ratios* | *CI* | *p* |  |
| SCZ PRS: Rare use, low potency | 1.24 | 0.56: 2.74 | 0.588 |  |
| SCZ PRS: Rare use, high potency | 1.12 | 0.45: 2.75 | 0.809 |  |
| SCZ PRS: Weekly use, low potency | 1.73 | 0.27: 1.94 | 0.524 |  |
| SCZ PRS: Weekly use, high potency | 2.23 | 0.45: 11.11 | 0.326 |  |
| SCZ PRS: Daily use, low potency | 0.66 | 0.21: 2.09 | 0.479 |  |
| SCZ PRS: Daily use, high potency | 0.78 | 0.26: 2.30 | 0.651 |  |

*S-Table 7 This reports the main effect of frequency-potency of cannabis use and of schizophrenia PRS, independent of each other on the risk of psychotic disorder, in the EU-GEI sample. All models were adjusted for age, sex recruitment site, tobacco smoking, and 10 principal components.*

|  | **Frequency/potency composite measure (N=1,468)** | | |
| --- | --- | --- | --- |
| *Predictors* | *Odds Ratios* | *CI* | *p* |
| Schizophrenia PRS | 2.89 | 2.31: 3.61 | **5.83x10^-20^** |
| Rare use, low potency | 0.84 | 0.58: 1.22 | 0.358 |
| Rare use, high potency | 1.52 | 0.98: 2.35 | 0.595 |
| Weekly use, low potency | 2.11 | 1.21: 3.69 | **8.97x10^-3^** |
| Weekly use, high potency | 3.06 | 1.60: 5.85 | **7.59x10^-4^** |
| Daily use, low potency | 3.02 | 1.80: 5.08 | **3.13x10^-5^** |
| Daily use, high potency | 5.09 | 3.08: 8.43 | **3.21x10^-10^** |

# *UK Biobank Replication Results*

Since we observed a significant association between schizophrenia PRS and cannabis use patterns in UK Biobank participants without psychosis only, we wanted to look more closely at these participants. We therefore investigated the relationship between schizophrenia PRS and cannabis initiation and/or patterns of cannabis use among UK Biobank controls with and without previous history of psychotic like experiences We observe some evidence that the effect size for schizophrenia PRS is reduced among subjects without PLE but given the differences in sample size this is hard to interpret. This will be something of interest for future research.

S-Table 8 The effect of schizophrenia PRS on patterns of cannabis use among UK Biobank controls with and without report of psychotic-like experiences. All models adjusted for age, sex, recruitment site, tobacco smoking, 10 principal components.

|  | **With PLE** | | | **Without PLE** | | |
| --- | --- | --- | --- | --- | --- | --- |
| *Predictors* | *Odds Ratios* | *CI* | *p* | *Odds Ratios* | *CI* | *p* |
| Schizophrenia PRS predicting lifetime use among controls | 1.11 | 1.04 – 1.19 | **<0.001** | 1.08 | 1.06 – 1.10 | **<0.001** |
| Observations | 6490 | | | 131018 | | |
| R^2^ Tjur |  |  |  |  |  |  |
| *Predictors* | *Odds Ratios* | *CI* | *p* | *Odds Ratios* | *CI* | *p* |
| Schizophrenia PRS predicting weekly use among controls | 1.04 | 0.91 – 1.18 | 0.58 | 1.09 | 1.05 – 1.13 | **<0.001** |
| Observations | 6241 | | | 128535 | | |
| R^2^ Tjur | 0.085 | | | 0.052 | | |
| *Predictors* | *Odds Ratios* | *CI* | *p* | *Odds Ratios* | *CI* | *p* |
| Schizophrenia PRS predicting daily use among controls | 1.05 | 0.86 – 1.28 | 0.65 | 1.16 | 1.09 – 1.23 | **<0.001** |
| Observations | 4592 | | | 105071 | | |
| R^2^ Tjur | 0.232 | | | 0.135 | | |

*The independent and combined effect of schizophrenia PRS and pattern of cannabis use on the odds ratio for Psychotic Disorder*

In the UK Biobank sample, lifetime cannabis use, weekly, and daily use were all associated with increased risk of psychotic disorder (lifetime: crude OR=1.56, 95% CI [1.32,1.85,], p=2.32x10^-^; adjusted OR= 1.54, 95% CI [1.30,1.82], p=6.17x10^-7^; weekly: crude OR=1.98, 95% CI [1.46,2.62], p=3.95x10^-6^; adjusted OR= 1.94, 95% CI [1.44,2.57], p=6.54x10^-6^; daily: crude OR=4.60; 95% CI [3.22,6.44], p=4.95x10^-18^; adjusted OR= 4.47, 95% CI [3.14,6.26], p=1.77x10^-17^) . We saw no evidence of an interaction between schizophrenia PRS and levels of cannabis use.

S-Table 9 The interaction between schizophrenia PRS and levels of cannabis use in UK Biobank sample

|  | **UKB** | | |
| --- | --- | --- | --- |
| *Predictors* | *Odds Ratios* | *CI* | *p* |
| Schizophrenia PRS | 1.28 | 1.16 – 1.42 | **1.72e-06** |
| Lifetime cannabis use | 1.51 | 1.27 – 1.78 | **2.18e-06** |
| Schizophrenia PRS:Lifetime cannabis use | 1.00 | 0.86 – 1.18 | 9.74e-01 |
| Observations | 142085 | | |
| R^2^ Tjur | 0.002 | | |
| *Predictors* | *Odds Ratios* | *CI* | *p* |
| Schizophrenia PRS | 1.31 | 1.19 – 1.44 | **2.24e-08** |
| Weekly cannabis use | 2.06 | 1.54 – 2.71 | **4.48e-07** |
| Schizophrenia PRS:Weekly cannabis use | 0.79 | 0.62 – 1.02 | 5.52e-02 |
| Observations | 139170 | | |
| R^2^ Tjur | 0.001 | | |
| *Predictors* | *Odds Ratios* | *CI* | *p* |
| Schizophrenia PRS | 1.28 | 1.15 – 1.42 | **6.12e-06** |
| Daily cannabis use | 4.08 | 2.84 – 5.74 | **4.10e-15** |
| Schizophrenia PRS:Daily cannabis use | 1.10 | 0.82 – 1.55 | 5.35e-01 |
| Observations | 113240 | | |
| R^2^ Tjur | 0.002 | | |

Although we have shown that schizophrenia PRS is not significantly associated with case/control status in the UK Biobank cohort, we show that daily weekly users have a higher probability of being a case, compared to less frequent users. Despite overlapping confidence intervals, S-Figure 12 suggests a trend for heightened risk among daily users as schizophrenia PRS increases.

S-Figure 12 Probability of psychosis case status as schizophrenia PRS increases, across five levels of cannabis frequency in the UK Biobank data.


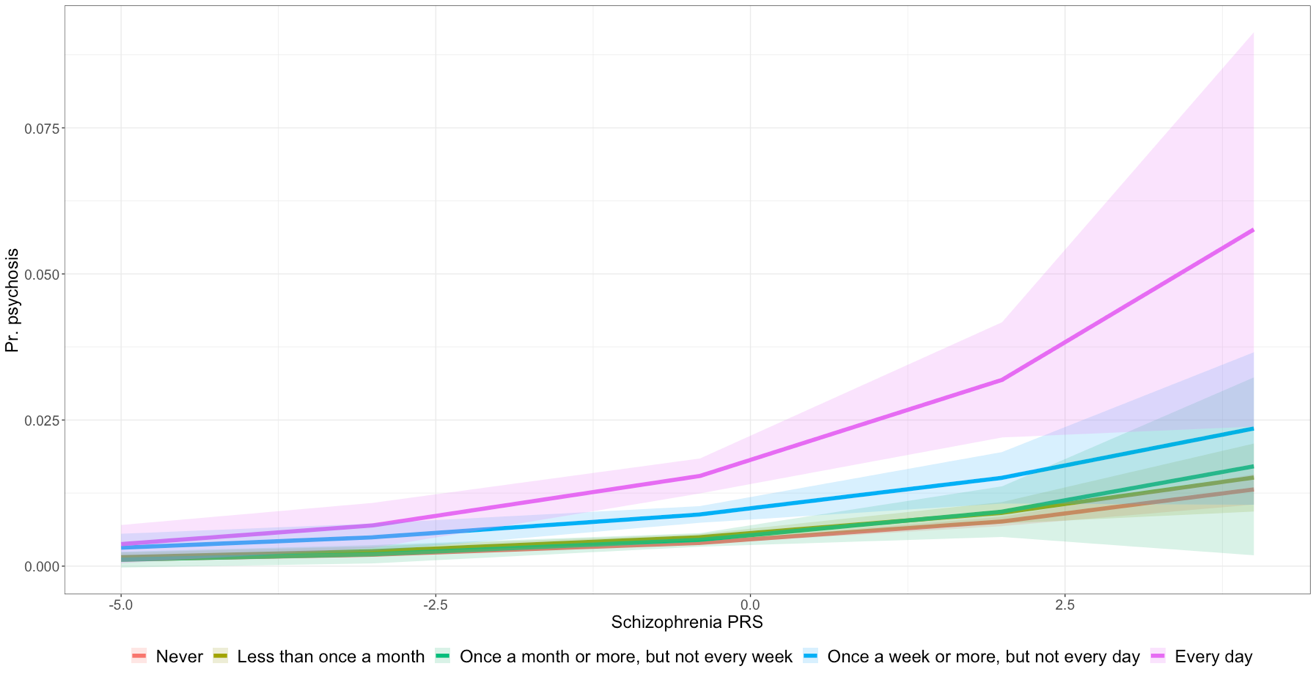


# Interaction Analyses: Additional Discussion

A paper by Matthew C. Keller (Keller, 2014) suggests that gene x environment interaction studies are not properly controlling for confounding. This paper specifically discusses candidate gene x environment interactions and talks of the issues of poor replication and corroboration by GWAS well known in candidate gene studies. Although our study uses PRS built on well-powered GWAS, and our results replicate in an independent sample (UKBB), the underlying statistical methodology can similarly be applied.

The interaction models in our study have three main terms: 1) schizophrenia PRS, 2) cannabis use pattern, and 3) the interaction between schizophrenia PRS and cannabis use pattern. Given the likelihood that both of these main predictor variables could be impacted by age, sex, ancestry, or location, we additionally adjust for these variables in the model. However, Keller states that although including these covariates does control for their potentially confounding influences on the main effects (PRS/cannabis), it does not control for the potential confounding these variables might cause on the interaction term (PRS x cannabis). Instead, Keller suggests that we should include all the covariate-by-environment (E) and the covariate-by-gene (G) interaction terms in the same model that tests the gene-by-environment interaction term. Thus, our model would be: case_control ~ PRS + cannabis_use + PRS x cannabis_use + age + sex + site + cigaret + PC1|PC10 + PRS x age + PRS x sex + PRS x site + PRS x cigaret + PRS x PC1|PRS x PC10 + cannabis_use x age + cannabis_use x sex + cannabis_use x site + cannabis_use x cigaret + cannabis_use x PC1|cannabis_use x PC10. We conducted this analysis and confirmed that there is no evidence of interaction between our primary variables schizophrenia in PRS and cannabis use in either cohort.

*S-Table 10 Assessing Keller additional interaction analyses*

|  |  | **EU-GEI** | | |
| --- | --- | --- | --- | --- |
| *Predictors* | *N* | *OR* | *CI* | *p* |
| Schizophrenia PRS: Lifetime cannabis use | 1,541 | 0.92 | 0.55 – 1.54 | 0.762 |
| Schizophrenia PRS: Weekly cannabis use | 1,297 | 1.80 | 0.65 – 6.04 | 0.293 |
| Schizophrenia PRS: Daily cannabis use | 1,396 | 0.72 | 0.33 – 1.62 | 0.413 |

|  |  | **UK Biobank** | | |
| --- | --- | --- | --- | --- |
| *Predictors* | *N* | *OR* | *CI* | *p* |
| Schizophrenia PRS: Lifetime cannabis use | 139,869 | 0.88 | 0.70 – 1.09 | 0.236 |
| Schizophrenia PRS: Weekly cannabis use | 137,024 | 0.80 | 0.56 – 1.17 | 0.249 |
| Schizophrenia PRS: Daily cannabis use | 111,408 | 0.89 | 0.57 – 1.43 | 0.632 |

*S-Table 11 Assessing relative excess risk of interaction (RERI)*

|  | OR (95% CI) | P value |
| --- | --- | --- |
| EU-GEI | 1.82 (-0.35:3.99) | 0.100 |
| UK Biobank | 0.171 (0.05:0.29) | 0.007 |

# ***Inclusion of Cannabis Use Disorder PRS to primary models***

Table 2 in the main manuscripts reports the results for schizophrenia PRS predicting cannabis use patterns in EU-GEI and UK Biobank cases and controls. We also investigated the role of CUD PRS in predicting patterns of cannabis use in our two cohorts, both when modelled independently and jointly with schizophrenia PRS.

In the EU-GEI sample, and in UK Biobank participants with psychosis the schizophrenia PRS is not predictive of cannabis use patterns in either cases or controls (Table 2). Inclusion of the CUS PRS alongside the schizophrenia PRS does not change this (S-Table 12, S-Table 14). In UK Biobank participants without psychosis, the schizophrenia PRS is predictive of lifetime, weekly and daily cannabis use. The effect size for schizophrenia PRS is reduced in additionally adjusted for CUD PRS, indicating the correlated CUD PRS explains some of this association (S-Table 14). A comparison of these models is shown in S-Figure 13 and S-Figure 14.

S-Table 12 Cannabis use disorder (CUD) PRS predicting patterns of cannabis use among EU-GEI cases and controls. All models adjusted for age, sex, site, and 10 principal components.

|  | **EU-GEI cases** | | | | | | | | | | | | **EU-GEI controls** | | | |
| --- | --- | --- | --- | --- | --- | --- | --- | --- | --- | --- | --- | --- | --- | --- | --- | --- |
| *Predictors* | *Odds Ratios* | | | *CI* | | | | | | *p* | | | *Odds Ratios* | | *CI* | *p* |
| CUD PRS predicting lifetime use | 1.57 | | | 1.03 – 2.40 | | | | | | **0.03** | | | 1.36 | | 1.00 – 1.86 | **0.05** |
| Observations | 628 | | | | | | | | | | | | 933 | | | |
| R^2^ Tjur | 0.306 | | | | | | | | | | | | 0.197 | | | |
|  | EU-GEI cases | | | | | | | | | | | | EU-GEI controls | | | |
| *Predictors* | *Odds Ratios* | | | *CI* | | | *p* | | | | | | *Odds Ratios* | | *CI* | *p* |
| CUD PRS predicting weekly use | 2.22 | | | 1.31 – 3.85 | | | **<0.01** | | | | | | 0.91 | | 0.49 – 1.68 | 0.76 |
| Observations | 450 | | | | | | | | | | | | 864 | | | |
| R^2^ Tjur | 0.199 | | | | | | | | | | | | 0.121 | | | |
|  | EU-GEI cases | | | | | | | | | | | | EU-GEI controls | | | |
| *Predictors* | | *Odds Ratios* | | | *CI* | | | *p* | | | *Odds Ratios* | | | | *CI* | *p* |
| CUD PRS predicting daily use | | 1.26 | | | 0.80 – 1.98 | | | 0.31 | | | 1.84 | | | | 1.05 – 3.28 | **0.04** |
| Observations | | 535 | | | | | | | | | 876 | | | | | |
| R^2^ Tjur | | 0.275 | | | | | | | | | 0.094 | | | | | |
|  | | EU-GEI cases | | | | | | | | | EU-GEI controls | | | | | |
| *Predictors* | | | *Estimates* | | | *CI* | | | *p* | | | *Estimates* | | *CI* | | *p* |
| CUD PRS predicting age at first use | | | -0.00 | | | -0.82 – 0.82 | | | 1.00 | | | -0.12 | | -0.96 – 0.71 | | 0.77 |
| Observations | | | 416 | | | | | | | | | 422 | | | | |
| R^2^ / R^2^ adjusted | | | 0.258 / 0.213 | | | | | | | | | 0.173 / 0.123 | | | | |

S-Table 13 Schizophrenia and CUD PRS predicting patterns of cannabis use among EU-GEI cases and controls. All models adjusted for age, sex, site, and 10 principal components.

|  | **EU-GEI cases** | | | | | | | | | | | | **EU-GEI controls** | | | |
| --- | --- | --- | --- | --- | --- | --- | --- | --- | --- | --- | --- | --- | --- | --- | --- | --- |
| *Predictors* | *Odds Ratios* | | | *CI* | | | | | | *p* | | | *Odds Ratios* | | *CI* | *p* |
| Schizophrenia PRS predicting lifetime use | 0.84 | | | 0.58 – 1.21 | | | | | | 0.34 | | | 1.08 | | 0.85 – 1.38 | 0.52 |
| CUD PRS predicting lifetime use | 1.62 | | | 1.06 – 2.49 | | | | | | **0.03** | | | 1.33 | | 0.97 – 1.84 | 0.08 |
| Observations | 628 | | | | | | | | | | | | 933 | | | |
| R^2^ Tjur | 0.308 | | | | | | | | | | | | 0.197 | | | |
|  | EU-GEI cases | | | | | | | | | | | | EU-GEI controls | | | |
| *Predictors* | *Odds Ratios* | | | *CI* | | | *p* | | | | | | *Odds Ratios* | | *CI* | *p* |
| Schizophrenia PRS predicting weekly use | 1.03 | | | 0.63 – 1.72 | | | 0.90 | | | | | | 1.20 | | 0.73 – 2.00 | 0.47 |
| CUD PRS predicting weekly use | 2.20 | | | 1.28 – 3.87 | | | **<0.01** | | | | | | 0.87 | | 0.46 – 1.62 | 0.65 |
| Observations | 450 | | | | | | | | | | | | 864 | | | |
| R^2^ Tjur | 0.199 | | | | | | | | | | | | 0.123 | | | |
|  | EU-GEI cases | | | | | | | | | | | | EU-GEI controls | | | |
| *Predictors* | | *Odds Ratios* | | | *CI* | | | *p* | | | *Odds Ratios* | | | | *CI* | *p* |
| Schizophrenia PRS predicting daily use | | 0.76 | | | 0.51 – 1.12 | | | 0.16 | | | 1.13 | | | | 0.72 – 1.77 | 0.60 |
| CUD PRS predicting daily use | | 1.34 | | | 0.85 – 2.11 | | | 0.21 | | | 1.78 | | | | 1.00 – 3.21 | 0.05 |
| Observations | | 535 | | | | | | | | | 876 | | | | | |
| R^2^ Tjur | | 0.276 | | | | | | | | | 0.095 | | | | | |
|  | | EU-GEI cases | | | | | | | | | EU-GEI controls | | | | | |
| *Predictors* | | | *Estimates* | | | *CI* | | | *p* | | | *Estimates* | | *CI* | | *p* |
| Schizophrenia PRS predicting age at first use | | | 0.10 | | | -0.64 – 0.85 | | | 0.78 | | | -0.23 | | -0.92 – 0.47 | | 0.52 |
| CUD PRS predicting age at first use | | | -0.03 | | | -0.88 – 0.81 | | | 0.94 | | | -0.04 | | -0.91 – 0.83 | | 0.92 |
| Observations | | | 416 | | | | | | | | | 422 | | | | |
| R^2^ / R^2^ adjusted | | | 0.258 / 0.211 | | | | | | | | | 0.174 / 0.121 | | | | |

S-Figure 13 Demonstrating the similarity in models with and without jointly modelled schizophrenia and CUD PRS in predicting cannabis use patterns in the EU-GEI sample.

***
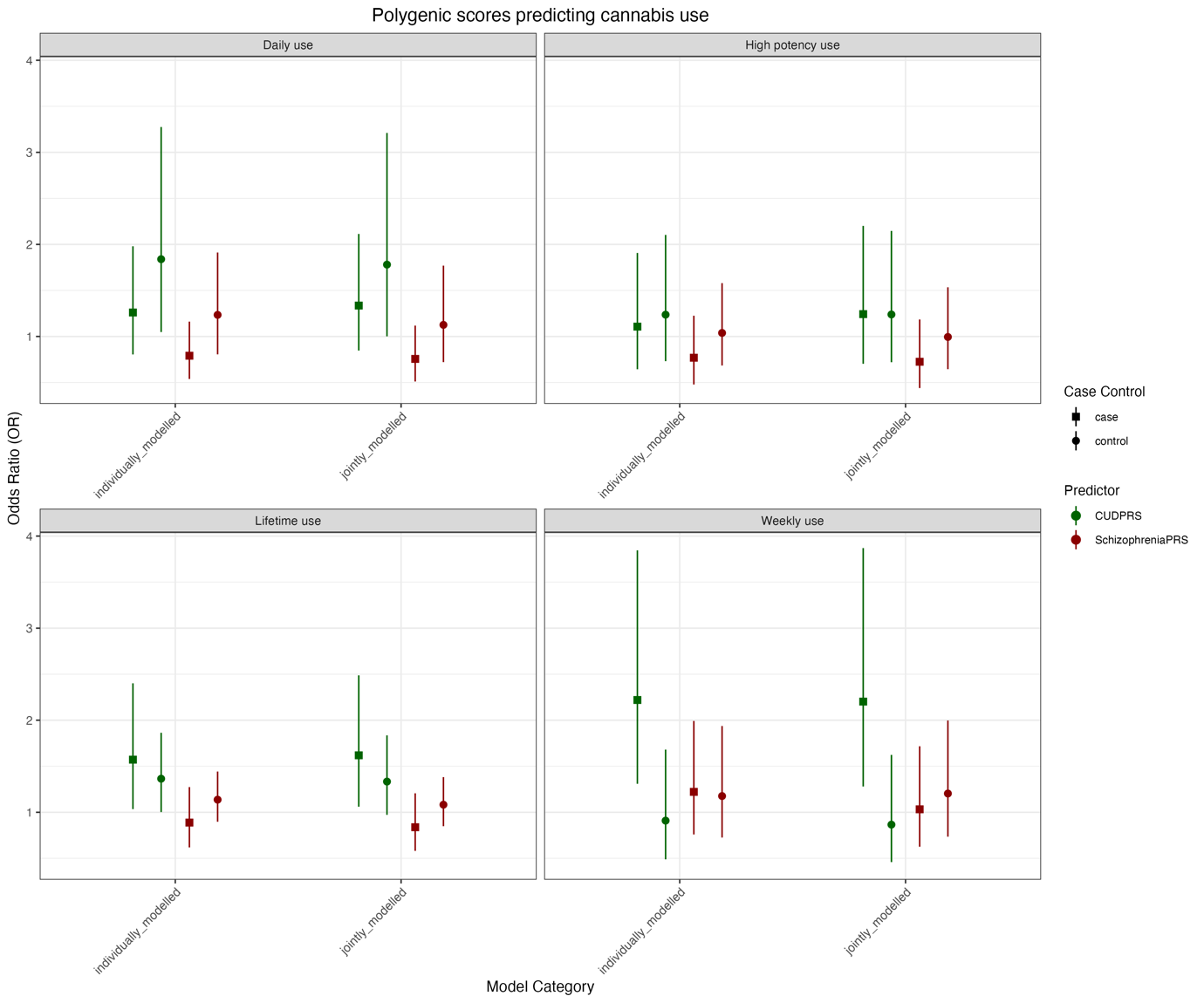
***

S-Table 14 Cannabis use disorder (CUD) PRS predicting patterns of cannabis use among UK Biobank participants with and without psychosis. All models adjusted for age, sex, site, genotype batch, and10 principal components.

|  | **UKB cases** | | | **UKB controls** | | |
| --- | --- | --- | --- | --- | --- | --- |
| *Predictors* | *Odds Ratios* | *CI* | *p* | *Odds Ratios* | *CI* | *p* |
| CUD PRS predicting lifetime use | 0.96 | 0.80 – 1.16 | 0.70 | 1.07 | 1.06 – 1.09 | **<0.01** |
| Observations | 759 | | | 141326 | | |
| R^2^ Tjur | 0.254 | | | 0.156 | | |

| *Predictors* | *Odds Ratios* | *CI* | *p* | *Odds Ratios* | *CI* | *p* |
| --- | --- | --- | --- | --- | --- | --- |
| CUD PRS predicting weekly use | 0.86 | 0.61 – 1.23 | 0.41 | 1.10 | 1.06 – 1.13 | **<0.01** |
| Observations | 706 | | | 138464 | | |
| R^2^ Tjur | 0.227 | | | 0.054 | | |

| *Predictors* | *Odds Ratios* | *CI* | *p* | *Odds Ratios* | *CI* | *p* |
| --- | --- | --- | --- | --- | --- | --- |
| CUD PRS predicting daily use | 1.01 | 0.67 – 1.55 | 0.95 | 1.21 | 1.14 – 1.27 | **<0.01** |
| Observations | 547 | | | 112693 | | |
| R^2^ Tjur | 0.427 | | | 0.146 | | |

S-Table 15 Schizophrenia and CUD PRS predicting patterns of cannabis use among UK Biobank controls with and without PLE. All models adjusted for age, sex, site, genotype batch, and10 principal components.

|  | **UKB cases** | | | **UKB controls** | | |
| --- | --- | --- | --- | --- | --- | --- |
| *Predictors* | *Odds Ratios* | *CI* | *p* | *Odds Ratios* | *CI* | *p* |
| Schizophrenia PRS predicting lifetime use | 0.95 | 0.75 – 1.20 | 0.66 | 1.06 | 1.04 – 1.08 | **<0.01** |
| CUD PRS predicting lifetime use | 0.99 | 0.80 – 1.21 | 0.89 | 1.05 | 1.03 – 1.06 | **<0.01** |
| Observations | 759 | | | 141326 | | |
| R^2^ Tjur | 0.255 | | | 0.156 | | |

| *Predictors* | *Odds Ratios* | *CI* | *p* | *Odds Ratios* | *CI* | *p* |
| --- | --- | --- | --- | --- | --- | --- |
| Schizophrenia PRS predicting weekly use | 0.93 | 0.60 – 1.44 | 0.73 | 1.06 | 1.02 – 1.10 | **0.01** |
| CUD PRS predicting weekly use | 0.89 | 0.60 – 1.31 | 0.55 | 1.07 | 1.03 – 1.11 | **<0.01** |
| Observations | 706 | | | 138464 | | |
| R^2^ Tjur | 0.227 | | | 0.055 | | |
| *Predictors* | *Odds Ratios* | *CI* | *p* | *Odds Ratios* | *CI* | *p* |
| Schizophrenia PRS predicting daily use | 1.05 | 0.61 – 1.86 | 0.86 | 1.05 | 0.98 – 1.12 | 0.14 |
| CUD PRS predicting daily use | 0.99 | 0.63 – 1.58 | 0.98 | 1.18 | 1.12 – 1.26 | **<0.01** |
| Observations | 547 | | | 112693 | | |
| R^2^ Tjur | 0.428 | | | 0.146 | | |

S-Figure 14 Demonstrating the similarity in models with and without jointly modelled schizophrenia and CUD PRS in predicting cannabis use patterns in the UK Biobank sample.


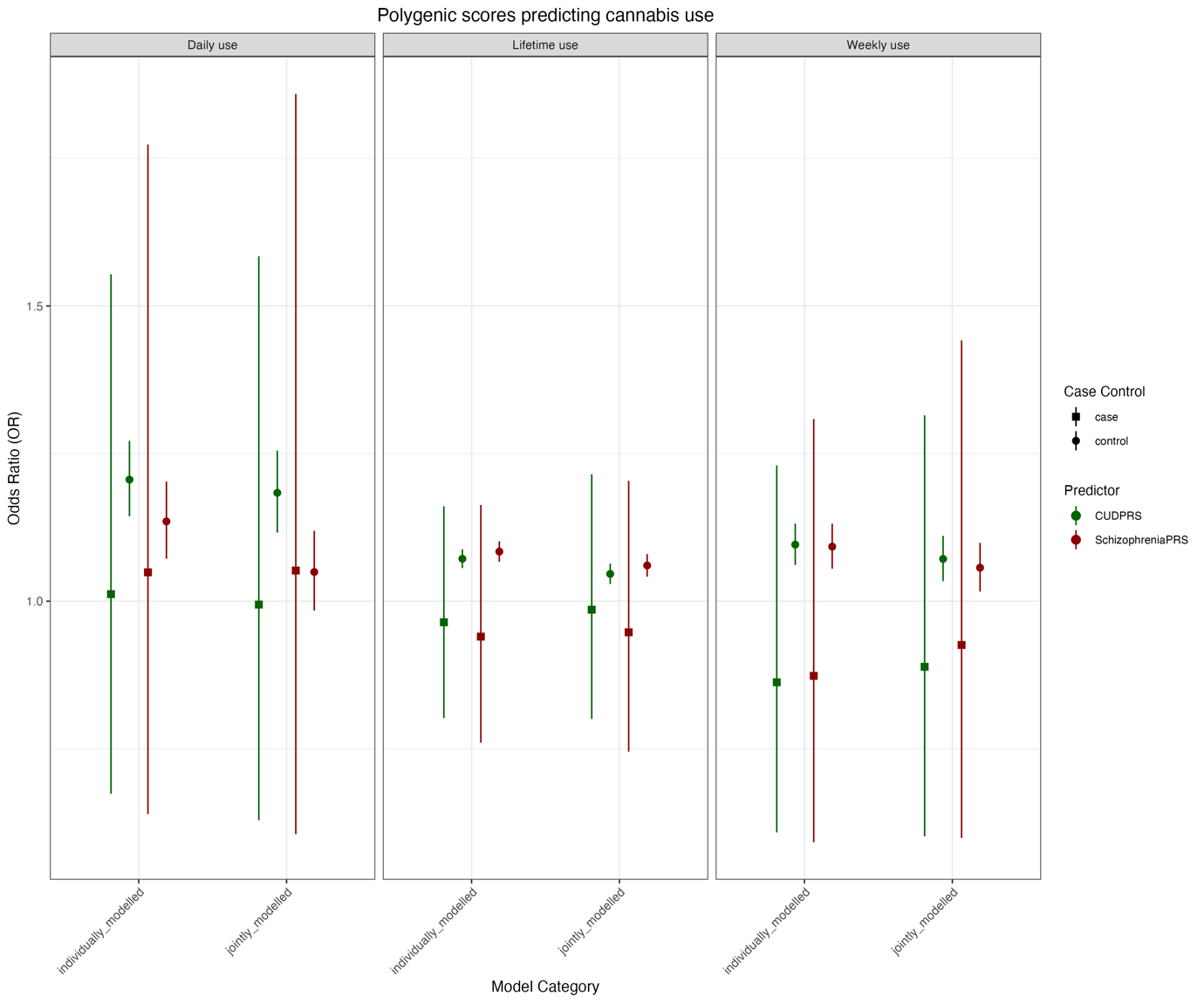


We also looked at the impact of additionally adjusting for CUD PRS in our models looking at schizophrenia PRS predicting cannabis use among UK Biobank controls with and without psychotic like experiences (PLEs). Schizophrenia PRS remained associated with cannabis use levels all levels of cannabis use in UK Biobank controls without history of PLE, however the effect sizes were reduced by >10% in all cases when CUD PRS was included in the models (S-Table 16).

S-Table 16 The effect of schizophrenia PRS on patterns of cannabis use among UK Biobank controls with and without report of psychotic-like experiences. All models adjusted for age, sex, recruitment site, tobacco smoking, 10 principal components.

|  | **With PLE** | | | **Without PLE** | | |
| --- | --- | --- | --- | --- | --- | --- |
| *Predictors* | *Odds Ratios* | *CI* | *p* | *Odds Ratios* | *CI* | *p* |
| Schizophrenia PRS predicting daily use among controls | 0.92 | 0.74 – 1.16 | 0.49 | 1.07 | 1.00 – 1.15 | **0.05** |
| CUD PRS predicting lifetime use among controls | 1.31 | 1.07 – 1.61 | **0.01** | 1.17 | 1.10 – 1.25 | **<0.01** |
| Observations | 4592 | | | 105071 | | |
| R^2^ Tjur | 0.235 | | | 0.137 | | |
| *Predictors* | *Odds Ratios* | *CI* | *p* | *Odds Ratios* | *CI* | *p* |
| Schizophrenia PRS predicting weekly use among controls | 1.00 | 0.87 – 1.15 | 1.00 | 1.06 | 1.01 – 1.10 | **0.01** |
| CUD PRS predicting lifetime use among controls | 1.08 | 0.95 – 1.22 | 0.27 | 1.07 | 1.03 – 1.11 | **<0.01** |
| Observations | 6241 | | | 128535 | | |
| R^2^ Tjur | 0.085 | | | 0.052 | | |
| *Predictors* | *Odds Ratios* | *CI* | *p* | *Odds Ratios* | *CI* | *p* |
| Schizophrenia PRS predicting daily use among controls | 0.92 | 0.74 – 1.16 | 0.49 | 1.07 | 1.00 – 1.15 | **0.05** |
| CUD PRS predicting lifetime use among controls | 1.31 | 1.07 – 1.61 | **0.01** | 1.17 | 1.10 – 1.25 | **<0.01** |
| Observations | 4592 | | | 105071 | | |
| R^2^ Tjur | 0.235 | | | 0.137 | | |

We also adjusted models investigating the combined impact of schizophrenia PRS and varying levels of cannabis use (lifetime, weekly, daily) in predicting case status. In all models within EU-GEI, we demonstrate that the CUD PRS is predicative of case status, even when adjusting for schizophrenia PRS and cannabis use frequency. However, there is little evidence that the CUD PRS is confounding the results for the schizophrenia PRS, as the effects are equivocal in models with and without the CUD PRS (S-Table 14, S-Figure 14). In the UK Biobank sample, CUD PRS is not associated with psychosis status, and again there is no evidence that CUD PRS confounds the observations for schizophrenia PRS and levels of cannabis use. (S-Table 15, S-Figure 15).

S-Table 17 Schizophrenia PRS, CUD PR, and measures of cannabis use independently predict FEP case status in the EU-GEI cohort

|  | **EU-GEI** | | |
| --- | --- | --- | --- |
| *Predictors* | *Odds Ratios* | *CI* | *p* |
| Schizophrenia PRS | 2.68 | 2.17 – 3.34 | **3.86e-19** |
| CUD PRS | 1.70 | 1.32 – 2.19 | **3.44e-05** |
| Lifetime cannabis use | 1.58 | 1.22 – 2.06 | **6.01e-04** |
| Observations | 1561 | | |
| R^2^ Tjur | 0.250 | | |

|  |  | | |
| --- | --- | --- | --- |
| *Predictors* | *Odds Ratios* | *CI* | *p* |
| Schizophrenia PRS | 2.77 | 2.19 – 3.52 | **4.34e-17** |
| CUD PRS | 1.78 | 1.35 – 2.35 | **4.26e-05** |
| Weekly cannabis use | 2.28 | 1.50 – 3.47 | **1.08e-04** |
| Observations | 1314 | | |
| R^2^ Tjur | 0.217 | | |

|  |  | | |
| --- | --- | --- | --- |
| *Predictors* | *Odds Ratios* | *CI* | *p* |
| Schizophrenia PRS | 2.70 | 2.15 – 3.41 | **3.45e-17** |
| CUD PRS | 1.51 | 1.15 – 1.98 | **3.00e-03** |
| Daily cannabis use | 3.53 | 2.46 – 5.10 | **1.04e-11** |
| Observations | 1411 | | |
| R^2^ Tjur | 0.267 | | |

S-Figure 14 Demonstrating the impact of additionally adjusting for CUD PRS in quantifying the independent and combined effects of polygenic scores and cannabis use patterns in the EU-GEI sample

***
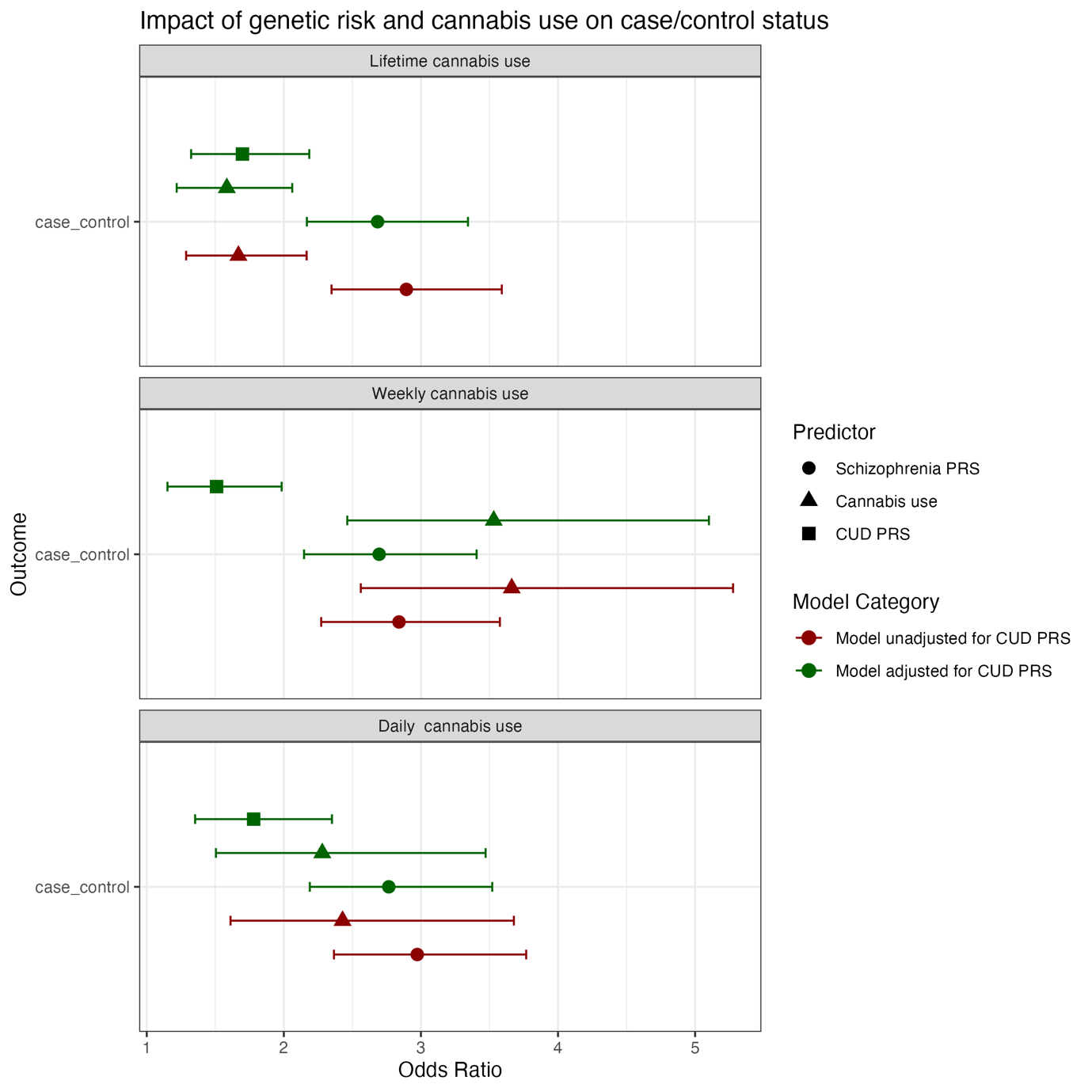
***

S-Table 18 Schizophrenia PRS and measures of cannabis use independently predict psychosis status in the UK Biobank cohort, both when adjusted for CUD PRS and not.

| *Predictors* | *Odds Ratios* | *CI* | *p* |
| --- | --- | --- | --- |
| Schizophrenia PRS | 1.31 | 1.19 – 1.44 | **4.16e-08** |
| CUD PRS | 0.96 | 0.88 – 1.05 | 4.08e-01 |
| Lifetime cannabis use | 1.51 | 1.27 – 1.78 | **1.43e-06** |
| Observations | 142085 | | |
| R^2^ Tjur | 0.002 | | |
| *Predictors* | *Odds Ratios* | *CI* | *p* |
| Schizophrenia PRS | 1.31 | 1.19 – 1.44 | **4.16e-08** |
| CUD PRS | 0.96 | 0.88 – 1.05 | 4.08e-01 |
| Lifetime cannabis use | 1.51 | 1.27 – 1.78 | **1.43e-06** |
| Observations | 142085 | | |
| R^2^ Tjur | 0.002 | | |
| *Predictors* | *Odds Ratios* | *CI* | *p* |
| Schizophrenia PRS | 1.32 | 1.18 – 1.47 | **1.66e-06** |
| CUD PRS | 0.95 | 0.86 – 1.05 | 3.42e-01 |
| Daily cannabis use | 4.19 | 2.95 – 5.85 | **2.09e-16** |
| Observations | 113240 | | |
| R^2^ Tjur | 0.002 | | |

S-Figure 17 Demonstrating the impact of additionally adjusting for CUD PRS in quantifying the independent and combined effects of polygenic scores and cannabis use patterns in the UK Biobank sample


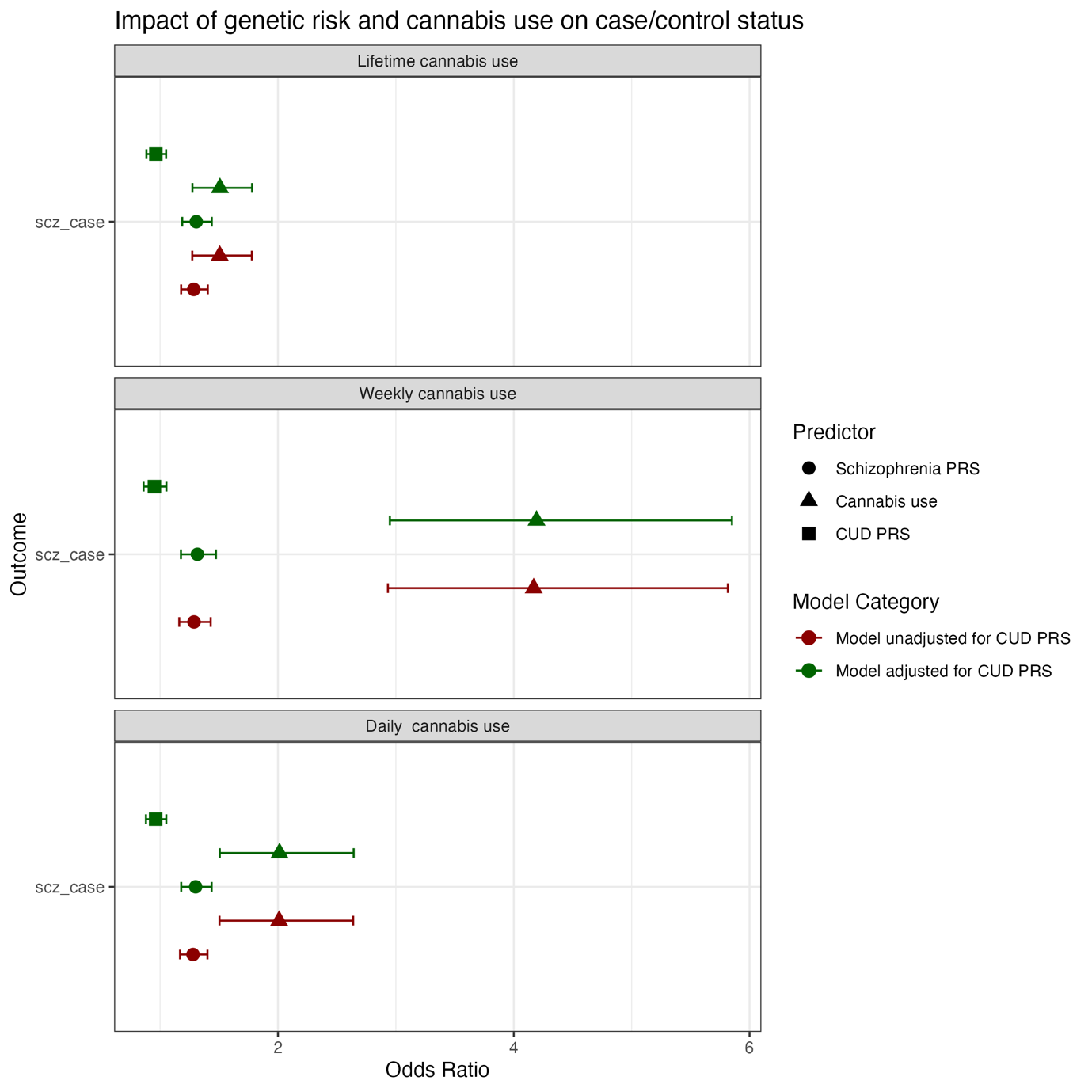


# *European Ancestry sensitivity analyses*

In the main text we report an analysis including participants of all genetic ancestry groups. However, individuals of predominantly European ancestry (EUR) make up the majority of the sample in both EU-GEI and UK Biobank, and polygenic risk scores are known to be more accurate for individuals of predominantly European ancestry. We therefore conducted a EUR-only sensitivity analysis to ensure our results in the full sample were equivocal.

We observed no differences in our inclusive analysis and our EUR only analysis in EU-GEI (S-Figure 16) or UK Biobank (S-Figure 17); all confidence intervals overlap almost entirely, indicating no meaningful difference in the effect of schizophrenia PRS or different levels of cannabis is apparent. Similarly, we see no meaningful difference in our models adjusted for CUD PRS, as shown in the right hand panels of S-Figure 16 and S-Figure 17.

S-Figure 16 Compares the effect of schizophrenia PRS and different levels of cannabis use predicting case/control status in the full EU-GEI sample (blue) compared to EUR-only EU-GEI sample (orange).


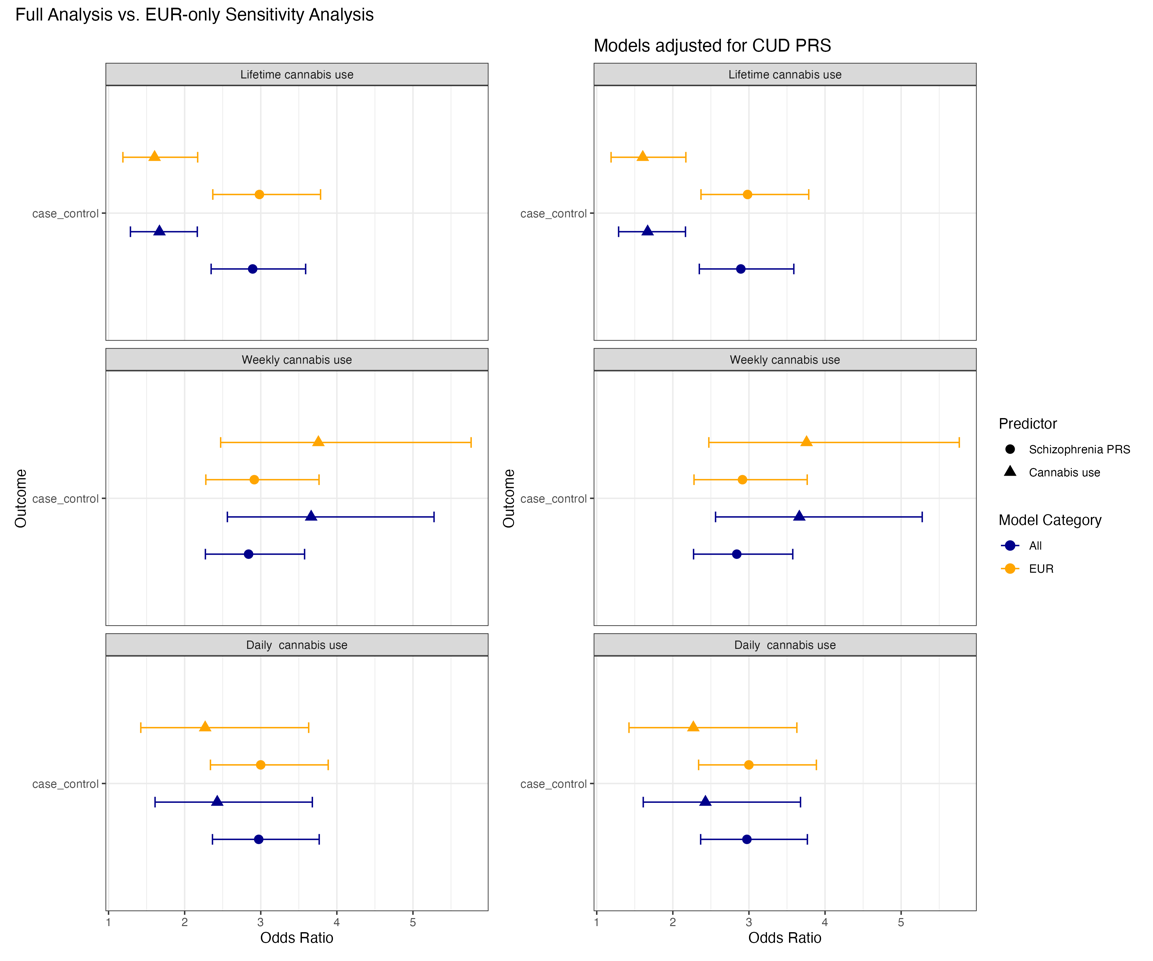


S-Figure 17 Compares the effect of schizophrenia PRS and different levels of cannabis use predicting psychosis status in the full UK Biobank sample (blue) compared to EUR-only UK Biobank sample (orange).


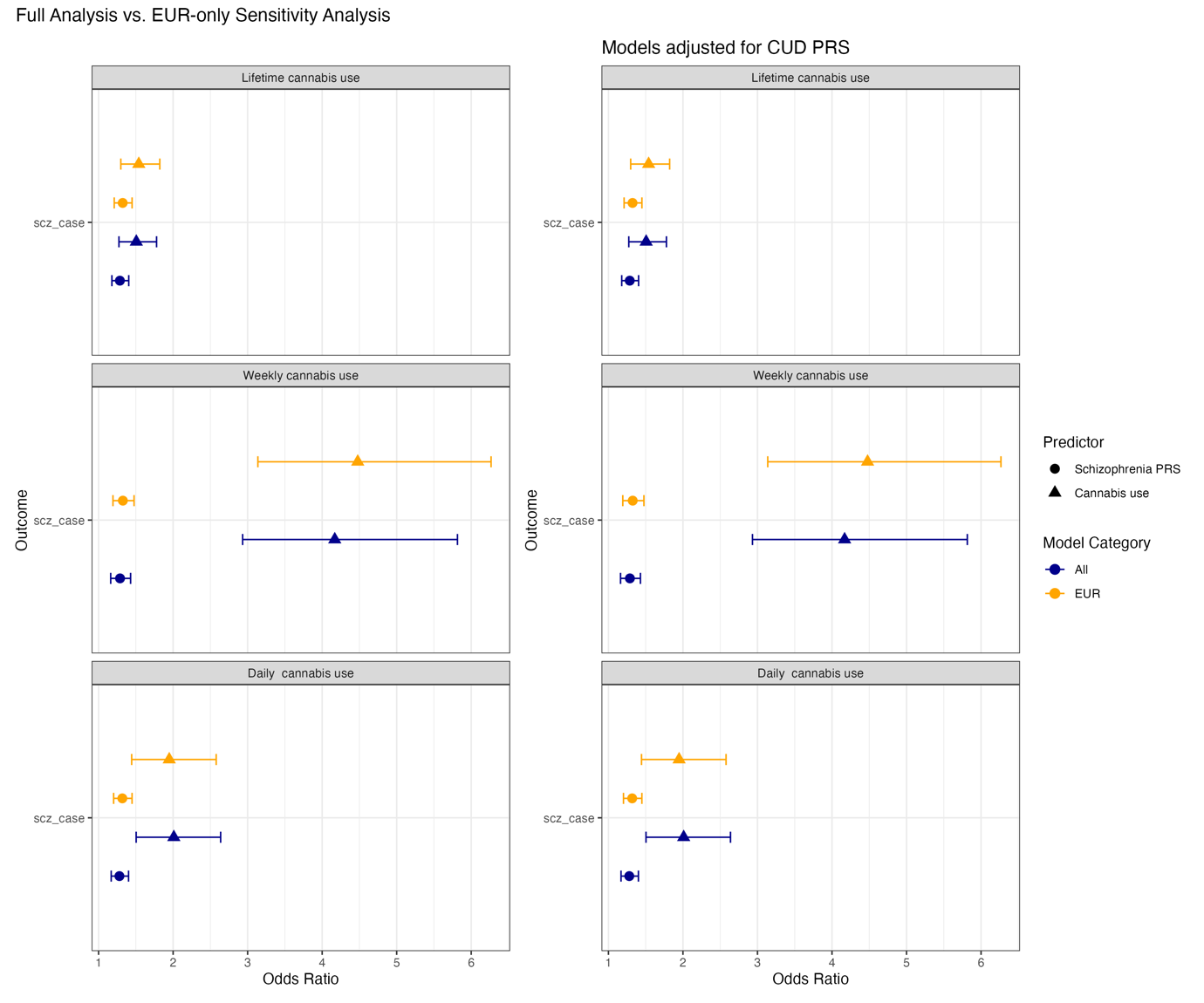


# EU-GEI WP2 Group

**EU-GEI COLLABORATORS**

Kathryn Hubbard^1^, Stephanie Beards^1,2^, Mara Parellada^3^, David Fraguas^3^, Marta Rapado Castro^3^, Álvaro Andreu-Bernabeu^3^, Gonzalo López^3^, Mario Matteis^3^, Emiliano González^3^, Manuel Durán-Cutilla^3^, Covadonga M. Díaz-Caneja^3^, Pedro Cuadrado^4^, José Juan Rodríguez Solano^5^, Angel Carracedo^6^, Javier Costas^6^, Emilio Sánchez^7^, Silvia Amoretti^8^, Esther Lorente-Rovira^9^, Paz Garcia-Portilla^10^, Estela Jiménez-López^11^, Eva Velthorst^12^, Nathalie Franke^12^, Daniella van Dam^12^, Fabian Termorshuizen^13,14^, Nathalie Franke^13^, Elsje van der Ven^13,14^, Elles Messchaart^14^, Marion Leboyer^15,16,17,18^, Stéphane Jamain^16,17,18^, Grégoire Baudin^15,16^, Aziz Ferchiou^15,16^, Baptiste Pignon^15,16,18^, Jean-Romain Richard^16,18^, Thomas Charpeaud^18,19,20^, Anne-Marie Tronche^18,19,20^, Pierre-Michel Llorca^20^, Flora Frijda^21,22^, Giovanna Marrazzo^22^, Lucia Sideli^22^, Crocettarachele Sartorio^22,23^, Fabio Seminerio^22^, Camila Marcelino Loureiro^24,25^, Rosana Shuhama^24,25^, Mirella Ruggeri^26^, Chiara Bonetto^26^, and Doriana Cristofalo^26^.

^1^Department of Health Service and Population Research, Institute of Psychiatry, Kings College London, De Crespigny Park, Denmark Hill, London SE5 8AF, UK.

^2^Department of Psychosis Studies, Institute of Psychiatry, King’s College London, De Crespigny Park, Denmark Hill, London SE5 8AF, UK.

^3^Department of Child and Adolescent Psychiatry, Hospital General Universitario Gregorio Marañón, School of Medicine, Universidad Complutense, IiSGM (CIBERSAM), C/Doctor Esquerdo 46, 28007 Madrid, Spain. ^4^Villa de Vallecas Mental Health Department, Villa de Vallecas Mental Health Centre, Hospital Universitario Infanta Leonor / Hospital Virgen de la Torre, C/San Claudio 154, 28038 Madrid, Spain.

^5^Puente de Vallecas Mental Health Department, Hospital Universitario Infanta Leonor / Hospital Virgen de la Torre, Centro de Salud Mental Puente de Vallecas, C/Peña Gorbea 4, 28018 Madrid, Spain.

^6^Fundación Pública Galega de Medicina Xenómica, Hospital Clínico Universitario, Choupana s/n, 15782 Santiago de Compostela, Spain.

^7^Department of Psychiatry, Hospital General Universitario Gregorio Marañón, School of Medicine, Universidad Complutense, IiSGM (CIBERSAM), C/Doctor Esquerdo 46, 28007 Madrid, Spain.

^8^Department of Psychiatry, Hospital Clinic, IDIBAPS, Centro de Investigación Biomédica en Red de Salud Mental (CIBERSAM), Universidad de Barcelona, C/Villarroel 170, escalera 9, planta 6, 08036 Barcelona, Spain.

^9^Department of Psychiatry, School of Medicine, Universidad de Valencia, Centro de Investigación Biomédica en Red de Salud Mental (CIBERSAM), C/Avda. Blasco Ibáñez 15, 46010 Valencia, Spain.

^10^Department of Medicine, Psychiatry Area, School of Medicine, Universidad de Oviedo, Centro de

Investigación Biomédica en Red de Salud Mental (CIBERSAM), C/Julián Clavería s/n, 33006 Oviedo, Spain. ^11^Department of Psychiatry, Servicio de Psiquiatría Hospital “Virgen de la Luz”, C/Hermandad de Donantes de Sangre, 16002 Cuenca, Spain.

^12^Department of Psychiatry, Early Psychosis Section, Academic Medical Centre, University of Amsterdam,

Meibergdreef 5, 1105 AZ Amsterdam, The Netherlands.

^13^Rivierduinen Centre for Mental Health, Leiden, Sandifortdreef 19, 2333 ZZ Leiden, The Netherlands. ^14^Department of Psychiatry and Neuropsychology, School for Mental Health and Neuroscience, South Limburg Mental Health Research and Teaching Network, Maastricht University Medical Centre, P.O. Box 616, 6200 MD Maastricht, The Netherlands.

^15^AP-HP, Groupe Hospitalier “Mondor”, Pôle de Psychiatrie, 51 Avenue de Maréchal de Lattre de Tassigny, 94010 Créteil, France.

^16^INSERM, U955, Equipe 15, 51 Avenue de Maréchal de Lattre de Tassigny, 94010 Créteil, France.

^17^Faculté de Médecine, Université Paris-Est, 51 Avenue de Maréchal de Lattre de Tassigny, 94010 Créteil, France.

^18^Fondation Fondamental, 40 Rue de Mesly, 94000 Créteil, France.

^19^CMP B CHU, BP 69, 63003 Clermont Ferrand, Cedex 1, France.

^20^Université Clermont Auvergne, EA 7280, Clermont-Ferrand 63000, France.

^21^Etablissement Public de Santé Maison Blanche, Paris, France.

^22^Department of Experimental Biomedicine and Clinical Neuroscience, Section of Psychiatry, University of Palermo, Via G. La Loggia n.1, 90129 Palermo, Italy.

^23^Unit of Psychiatry, “P. Giaccone” General Hospital, Via G. La Loggia n.1, 90129 Palermo, Italy. ^24^Departamento de Neurociências e Ciencias do Comportamento, Faculdade de Medicina de Ribeirão Preto, Universidade de São Paulo, Av. Bandeirantes, 3900 -Monte Alegre- CEP 14049-900, Ribeirão Preto, SP, Brasil.

^25^Núcleo de Pesquina em Saúde Mental Populacional, Universidade de São Paulo, Avenida

Doutor Arnaldo 455, CEP 01246-903, Ribeirão Preto, SP, Brasil.

^26^Section of Psychiatry, Department of Neuroscience, Biomedicine and Movement, University of Verona, Piazzale L. A. Scuro 10, 37134 Verona, Italy.

**References**

Brisacier, A.-C., Cadet-Taïrou, A., Díaz Gómez, C., Gandilhon, M., Le Nézet, O. & Lemenier-Jeannet, A. (2015). Drogues, chiffres clés. *Paris: Observatoire Français des Drogues et des Toxicomanies*. doi: Retrieved from <https://www.ofdt.fr/publications/collections/drogues-et-addictions-chiffres-cles/6eme-edition-2015/>.

Bycroft, C., Freeman, C., Petkova, D., Band, G., Elliott, L. T., Sharp, K., . . . O’Connell, J. (2018). The UK Biobank resource with deep phenotyping and genomic data. *Nature,* 562, 203-209. doi: 10.1038/s41586-018-0579-z.

de Oliveira, G. L., Voloch, M. H., Sztulman, G. B., Neto, O. N. & Yonamine, M. (2008). Cannabinoid contents in cannabis products seized in São Paulo, Brazil, 2006–2007. *Forensic Toxicology,* 26, 31-35. doi: 10.1007/s11419-008-0046-x.

Di Forti, M., Marconi, A., Carra, E., Fraietta, S., Trotta, A., Bonomo, M., . . . Russo, M. (2015). Proportion of patients in south London with first-episode psychosis attributable to use of high potency cannabis: a case-control study. *Lancet Psychiatry,* 2, 233-238. doi: 10.1016/S2215-0366(14)00117-5.

Di Forti, M., Quattrone, D., Freeman, T. P., Tripoli, G., Gayer-Anderson, C., Quigley, H., . . . La Cascia, C. (2019). The contribution of cannabis use to variation in the incidence of psychotic disorder across Europe (EU-GEI): a multicentre case-control study. *Lancet Psychiatry,* 6, 427-436. doi: 10.1016/S2215-0366(19)30048-3.

European Monitoring Centre for Drugs and Drug and Addiction (EMCDDA) (2016). European Drug Report 2016: Trends and Developments. Publications Office of the European Union. Luxembourg, 2016.

Fearon, P., Kirkbride, J. B., Morgan, C., Dazzan, P., Morgan, K., Lloyd, T., . . . Holloway, J. (2006). Incidence of schizophrenia and other psychoses in ethnic minority groups: results from the MRC AESOP Study. *Psychological medicine,* 36, 1541-1550. doi: Retrieved from

Ge, T., Chen, C.-Y., Ni, Y., Feng, Y.-C. A. & Smoller, J. W. (2019). Polygenic prediction via Bayesian regression and continuous shrinkage priors. *Nature Communications,* 10, 1776. doi: 10.1038/s41467-019-09718-5.

Ge, T., Irvin, M. R., Patki, A., Srinivasasainagendra, V., Lin, Y. F., Tiwari, H. K., . . . Karlson, E. W. (2022). Development and validation of a trans-ancestry polygenic risk score for type 2 diabetes in diverse populations. *Genome Medicine,* 14, 70. doi: 10.1186/s13073-022-01074-2.

Hardwick, S. & King, L. A. (2008). Home Office cannabis potency study 2008. doi: Retrieved from

Jongsma, H. (2018). The role of the sociocultural context in explaining variance in incidence of psychosis and higher rates of disorder in minorities. University of Cambridge.

Jongsma, H. E., Gayer-Anderson, C., Lasalvia, A., Quattrone, D., Mulè, A., Szöke, A., . . . Tarricone, I. (2018). Treated incidence of psychotic disorders in the multinational EU-GEI study. *JAMA Psychiatry,* 75, 36-46. doi: 10.1001/jamapsychiatry.2017.3554.

Keller, M. C. (2014). Gene x environment interaction studies have not properly controlled for potential confounders: the problem and the (simple) solution. *Biological Psychiatry,* 75, 18-24. doi: 10.1016/j.biopsych.2013.09.006.

Lee, S. H., Goddard, M. E., Wray, N. R. & Visscher, P. M. (2012). A better coefficient of determination for genetic profile analysis. *Genetic epidemiology,* 36, 214-24. doi: 10.1002/gepi.21614.

Lewis, C. M. & Vassos, E. (2017). Prospects for using risk scores in polygenic medicine. *Genome Medicine,* 9, 96. doi: 10.1186/s13073-017-0489-y.

McGuffin, P., Farmer, A. & Harvey, I. (1991). A polydiagnostic application of operational criteria in studies of psychotic illness: development and reliability of the OPCRIT system. *Archives of General Psychiatry,* 48, 764-770. doi: 10.1001/archpsyc.1991.01810320088015.

Niesink, R. J., Rigter, S., Koeter, M. W. & Brunt, T. M. (2015). Potency trends of Δ9‐tetrahydrocannabinol, cannabidiol and cannabinol in cannabis in the Netherlands: 2005–15. *Addiction,* 110, 1941-1950. doi: 10.1111/add.13082.

Pasman, J. A., Verweij, K. J., Gerring, Z., Stringer, S., Sanchez-Roige, S., Treur, J. L., . . . Ong, J.-S. (2018). GWAS of lifetime cannabis use reveals new risk loci, genetic overlap with psychiatric traits, and a causal effect of schizophrenia liability. *Nature Neuroscience,* 21, 1161-1170. doi: 10.1038/s41593-018-0206-1.

Potter, D. J., Clark, P. & Brown, M. B. (2008). Potency of delta 9-THC and other cannabinoids in cannabis in England in 2005: implications for psychoactivity and pharmacology. *Journal of Forensic Sciences,* 53, 90-4. doi: 10.1111/j.1556-4029.2007.00603.x.

Potter, D. J., Hammond, K., Tuffnell, S., Walker, C. & Di Forti, M. (2018). Potency of Delta(9) -tetrahydrocannabinol and other cannabinoids in cannabis in England in 2016: Implications for public health and pharmacology. *Drug Testing and Analysis,* 10, 628-635. doi: 10.1002/dta.2368.

Purcell, S., Neale, B., Todd-Brown, K., Thomas, L., Ferreira, M. A., Bender, D., . . . Daly, M. J. (2007). PLINK: a tool set for whole-genome association and population-based linkage analyses. *American Journal of Human Genetics,* 81, 559-575. doi: 10.1086/519795.

Quattrone, D., Di Forti, M., Gayer-Anderson, C., Ferraro, L., Jongsma, H. E., Tripoli, G., . . . Berardi, D. (2019). Transdiagnostic dimensions of psychopathology at first episode psychosis: findings from the multinational EU-GEI study. *Psychological Medicine,* 49, 1378-1391. doi: 10.1017/S0033291718002131.

Quattrone, D., Reininghaus, U., Richards, A. L., Tripoli, G., Ferraro, L., Quattrone, A., . . . Gayer-Anderson, C. (2021). The continuity of effect of schizophrenia polygenic risk score and patterns of cannabis use on transdiagnostic symptom dimensions at first-episode psychosis: findings from the EU-GEI study. *Translational Psychiatry,* 11, 423. doi: 10.1038/s41398-021-01526-0.

Ripke, S., Neale, B. M., Corvin, A., Walters, J. T., Farh, K.-H., Holmans, P. A., . . . Huang, H. (2014). Biological insights from 108 schizophrenia-associated genetic loci. *Nature*. doi: Retrieved from

Ruan, Y., Lin, Y. F., Feng, Y. A., Chen, C. Y., Lam, M., Guo, Z., . . . Ge, T. (2022). Improving polygenic prediction in ancestrally diverse populations. *Nature Genetics,* 54, 573-580. doi: 10.1038/s41588-022-01054-7.

Schizophrenia Working Group of the Psychiatric Genomics Consortium, R., S., Walters, J. T., & O’Donovan, M. C. (2020). Mapping genomic loci prioritises genes and implicates synaptic biology in schizophrenia. *MedRxiv*, 2020.09. 12.20192922. doi: Retrieved from

Vassos, E., Di Forti, M., Coleman, J., Iyegbe, C., Prata, D., Euesden, J., . . . Breen, G. (2017). An Examination of Polygenic Score Risk Prediction in Individuals With First-Episode Psychosis. *Biological Psychiatry,* 81, 470-477. doi: <https://doi.org/10.1016/j.biopsych.2016.06.028>.

Zamengo, L., Frison, G., Bettin, C. & Sciarrone, R. (2014). Cannabis potency in the Venice area (Italy): update 2013. *Drug Testing and Analysis,* 7, 255-258. doi: 10.1002/dta.1690.
